# Methyltetrahydrofolate vs Folic Acid Supplementation in Idiopathic Recurrent Miscarriage with Respect to Methylenetetrahydrofolate Reductase C677T and A1298C Polymorphisms: A Randomized Controlled Trial

**DOI:** 10.1371/journal.pone.0143569

**Published:** 2015-12-02

**Authors:** Azita Hekmatdoost, Farhad Vahid, Zahra Yari, Mohammadreza Sadeghi, Hassan Eini-Zinab, Niknam Lakpour, Soheila Arefi

**Affiliations:** 1 Department of Clinical Nutrition and Dietetics, Faculty of Nutrition and Food Technology, National Nutrition and Food Technology, Research Institute Shahid Beheshti University of Medical Sciences, Tehran, Iran; 2 National Nutrition and Food Technology Research Institute, Faculty of Nutrition and Food Technology, Students’ Research Committee, Shahid Beheshti University of Medical Sciences, Tehran, Iran; 3 Department of Andrology and Embryology, Reproductive Biotechnology Research Center, Avicenna Research Institute, ACECR, Tehran, Iran; 4 Department of Community Nutrition, Faculty of Nutrition and Food Technology, National Nutrition and Food Technology, Research Institute Shahid Beheshti University of Medical Sciences, Tehran, Iran; 5 Department of Endocrinology, Reproductive Biotechnology Research Center, Avicenna Research Institute, ACECR, Tehran, Iran; Aichi Cancer Center Research Institute, JAPAN

## Abstract

**Purpose:**

To determine whether 5-methylenetetrahydrofolate (MTHF) is more effective than folic acid supplementation in treatment of recurrent abortion in different MTHFR gene C677T and A1298C polymorphisms.

**Methods:**

A randomized, double blind, placebo-controlled trial conducted April 2011-September 2014 in recurrent abortion clinics in Tehran, Iran. The participants were women with three or more idiopathic recurrent abortion, aged 20 to 45 years. Two hundred and twenty eligible women who consented to participate were randomly assigned to receive either folic acid or 5-MTHF according to the stratified blocked randomization by age and the number of previous abortions. Participants took daily 1 mg 5-methylentetrahydrofolate or 1 mg folic acid from at least 8 weeks before conception to the 20^th^ week of the pregnancy. The primary outcome was ongoing pregnancy rate at 20^th^ week of pregnancy, and the secondary outcomes were serum folate and homocysteine at the baseline, after 8 weeks, and at the gestational age of 4, 8, 12, and 20 weeks, MTHFR gene C677T and A1298C polymorphisms.

**Results:**

There was no significant difference in abortion rate between two groups. Serum folate increased significantly in both groups over time; these changes were significantly higher in the group receiving 5-MTHF than the group receiving folic acid (value = 2.39, p<00.1) and the result was the same by considering the time (value = 1.24, p<0.01). Plasma tHcys decreased significantly in both groups over time; however these changes were not significantly different between the groups (value = 0.01, p = 0.47).

**Conclusion:**

The results do not support any beneficial effect of 5-MTHF vs. folate supplementation in women with recurrent abortion with any MTHFR C677T and/or A1298C polymorphism.

**Trial Registration:**

ClinicalTrials.gov NCT01976676

## Introduction

Idiopathic recurrent abortion (IRA) is a frustrating situation for the family, which affects 3% of the couples[1]. More than 20% of women with IRA have been noted to display elevated serum levels of total homocysteine (tHcy)[2, 3], and Low plasma folate levels were associated with an increased risk of early spontaneous abortion [4–8]. Disturbed methionine-homocysteine metabolism resulting in mild hyperhomocysteinemia has been reported as a risk factor for IRA[9–13] Therefore, lowering plasma tHcy concentrations is one of the most important therapeutic goals in IRA management, which usually overcome by folate supplementation [14–17].

On the other hand, polymorphisms associated with an impaired folate metabolism such as methyltetrahydrofolate reductase (MTHFR) C677T and A1298C are overrepresented among women with IRA [18, 19]. It seems that patients with mutated MTHFR gene respond to MTHF better than Folic acid supplementation. Thus, we designed this study to evaluate whether patients with MTHFR gene polymorphism benefit from administration of MTHF more than folic acid.

## Methods

### Study Design and Participants

In this stratified randomized double blind clinical trial, the patients were recruited between April 2011 and February 2014 from Recurrent Abortion clinics in Tehran, Iran. Eligible patients were informed about the study procedures by their gynecologist or trained clinic staffs. After a full review of the inclusion and exclusion criteria and explanation of the risks and benefits of the study, women with three or more idiopathic abortion who filled out the written consent form, were enrolled. All women underwent a standard diagnostic workup to rule out the presence of antiphospholipid syndrome or anatomic, cytogenetic, hormonal, or infectious pathologies. Diagnostic procedures included hysteroscopy; paternal and maternal karyotype; cervical cultures; a comprehensive hormonal status, and evaluation of antiphospholipid syndrome with IgM and IgG anticardiolipin antibody assessment and lupus anticoagulant testing.

Participants’ self-described age, race ethnicity, demographic information, medical, obstetric, occupational and family histories, lifetime history of tobacco use, intake of nutritional supplements, lifestyle and environmental exposures were recorded for preplanned analyses of possible moderators of the primary outcome.

### Ethical Approval

This trial was approved by the Human Ethics Committee of the National Nutrition and Food Technology Research Institute of Shahid Beheshti University of Medical Sciences (Ethics Code: 1157, June 2010). Written informed consent was obtained from all participants. This clinical trial was registered on IRCT (registration number: IRCT138903034010N1, July2010), and then, we also, registered it on clinicaltrial.gov (Identifier: NCT01976676, Oct 2013) because we thought that this registration web site is acceptable for a wide range of journals. The authors confirm that all ongoing and related trials for this drug/intervention are registered.

### Randomization, Masking and Interventions

**Fig 1:**
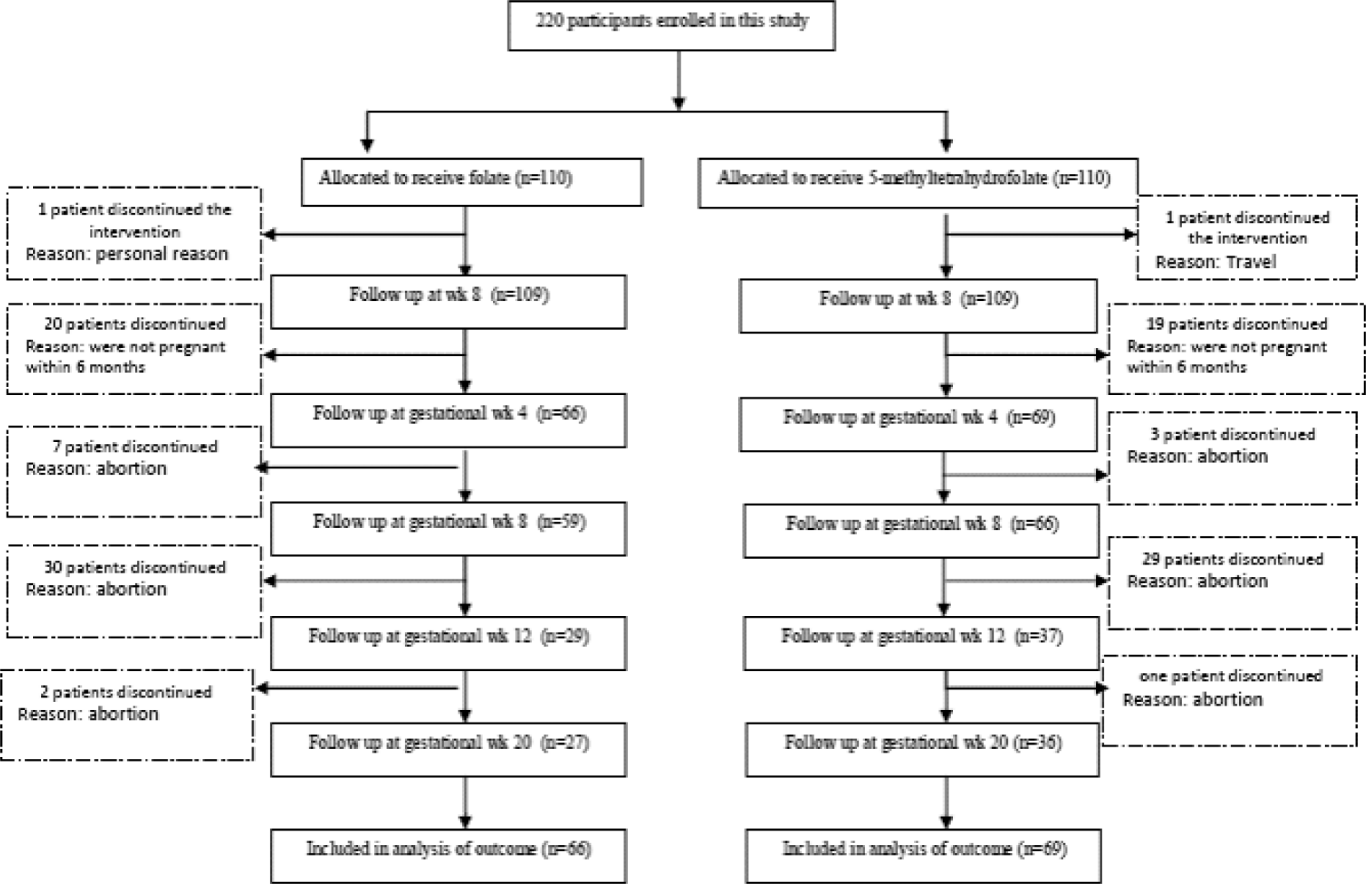
CONSORT Flow Diagram of Trial Participants.

Details about the recruitment, randomization, and follow-up of this study appear in the study flow diagram (Fig 1). Randomization was stratified by age and the number of abortions. Participants were assigned using computer-generated randomization to receive either 1mg folic acid or 1 mg 5-MTHF during the study period. The dosage was determined according to previous studies [20, 21], and every patient undertook her concurrent therapy along with the supplements. The supplements were purchased from Thorne Research Company, ID, USA. The group assignments were concealed in sealed envelopes and opened at enrollment by somebody who was blinded to all baseline assessments.

### Treatment and Follow-Up

The participants were evaluated by their Gynecologist at baseline, after 8 weeks, and at the gestational age of 4, 8, 12, and 20 weeks; women who did not get pregnant within six months of the study beginning, were excluded from the study. Study protocol adherence and plasma folate and homocystein concentration were assessed at all of these visits while plasma vitamin B_12_, Pyridoxal phosphate (PLP), and MTHFR gene polymorphisms for C677T and A1298C were evaluated at randomization time. Weekly telephone contacts were made to encourage adherence, and answer study-related questions. Adverse effects, adverse events, and medical status were recorded at each contact. The supplements were delivered to the patients every four weeks.

### Treatment Adherence

Every four weeks, participants were given enough supplements to last 3 days after their next scheduled visit and were instructed to return all unused supplements at each visit. The remaining supplements were counted and subtracted from the number provided to determine the number taken. The participants were asked to confirm that all capsules removed were actually taken as prescribed.

### Primary and Secondary Outcomes

The ongoing pregnancy at week 20^th^ was the primary outcome measure. Secondary outcomes included serum folate and tHcy concentration at the beginning, after 8 weeks, and at the gestational age of 4, 8, 12, and 20 week.

### Procedures

Information on baseline characteristics was obtained by interviewer administered questionnaire at the first visit and included data on demographics, occupation, medical history, number of previous abortions, smoking, and current medications. Folate, tHcy, Vitamin B_12,_ and PLP concentrations in EDTA-treated plasma were measured in EDTA-treated plasma with using their commercially available immunoassay kits for the IMx Analyzer (Abbott,Wiesbaden, Germany) with intraassay CV: <2.9%; interassay CV: <4.8%.

For assessment of *MTHFR gene polymorphism*, DNA was extracted from the leukocyte-rich buffy coat fraction of centrifuged, EDTA-treated whole blood by using the QIAamp DNA blood mini kit (Qiagen,Hilden, Germany); the MTHFR polymorphisms were detected according to the method of Zetterberg et al [22]. All other blood variables were measured with the use of standard automated laboratory techniques.

### Anthropometric Measurements

All participants underwent measurements of weight and height. Weight was measured to the nearest 0.5 kg using digital scales (Soehne, Berlin, Germany) with participants minimally clothed and barefoot. Height was measured to the nearest 0.5 cm using a fixed, non-stretch tape meter with participants barefoot in a standing position. Measurements were taken at the time of randomization. Body Mass Index (BMI) was calculated by dividing the patient’s weight in kilogram by his squared height in meter.

### Dietary Intake

Patients received prospective serial assessment of nutritional intake with three days written food records. All enrolled subjects received instructions to record their daily dietary intake for three days including a weekend day at the first, third, and last visits. Dietary intakes were analyzed by USDA food composition table (FCT) and Iranian FCT, and folate intakes from dietary sources were determined.

### Statistical Analysis

The sample size was calculated so that the 10 nmol/L difference in plasma folate indicates a significant difference, with power = 80. The sample size was calculated as 22 patients in each group. We included ten times more samples to outcome the loss to follow up in this study due to our previous experience at the clinic.

All continuous data were tested for normal distribution with the Shapiro–Wilk test. Parametric tests were used based on normal distributions, and nonparametric tests were performed when assumptions were not met.

Data analysis could be performed by repeated measure analysis of variance and intention-to-treat approach, but this method involves data imputation for missing ones which is not precise and suitable, so we selected mixed models and multivariate regression.

For data analysis, we converted single leveled data to multileveled data in order to run mixed regression models. Time and squared time are included in the model as the effect of time assumes to be quadratic.

Mixed models regression analysis was conducted to examine the relationship between serums folate or tHcy and various potential predictors after adjustment for baseline values and other confounders.

All hypothesis tests were two-tailed, with P<0.05 denoting statistical significance. SPSS version 20 and R statistical software version 3.1.2 were used for all statistical analyses.

## Results

Out of 220 participants, 135 women became pregnant within 6 months; twenty seven of 66 women in folic acid group and thirty six of 69 women in 5-MTHF group had ongoing pregnancy at the end of the study. Patient enrollment and retention by treatment group are shown in Fig 1. The baseline clinical and demographic data of the two groups were similar with respect to anthropometric data, laboratory data, dietary folate intake, and MTHFR polymorphism (Table 1).

**Table 1:** Demographic characteristics of the study sample at baseline.

Characteristics	Folic acid group (n = 110)	MTHF group (n = 110)
Age	33.4±4.5	33.6±5.1
BMI (kg/m^2^)	28.8 ±3.4	29.1±4.2
Dietary folate intake (μg/day)	246±32	250±28
Previous abortions	3.5±0.7	3.6±0.8
Plasma folate (nmol/L)	17.5±2.1	17.9±1.5
Plasma tHcys (μmol/L)	8.6±2.2	8.5±2.4
Plasma vit B12 (pmol/L)	234±102	240±111
MTHFR 677CT (n)		
C/C	55	54
C/T	44	45
T/T	11	11
MTHFR 1298AC (n)		
A/A	76	76
A/C	25	24
C/C	9	10

MTHFR, 5Methyltetrahydrofolate; BMI, Body Mass Index.

There was no significant difference between groups.

There was no significant difference in abortion rate between two groups. Furthermore, there was no significant relationship between abortion rate and participants’ MTHR genotypes, age, and number previous abortions; however, there was a significant association between abortion rate and plasma tHcys concentrations in 1^st^, 2^nd^, and 3^rd^ measurement.

With respect to dietary folate intake, no change was observed throughout the study period, so that it did not differ within two groups or between the groups at the first, third and last visits. Both supplements were well tolerated and safe; no treatment-related adverse side effects were observed. The compliance with respect to supplement intake was high and did not differ significantly between the groups (P > 0.05). All participants had consumed more than 90% of the supplements in each visit.

Values of the investigated clinical chemistry or hematologic blood variables before and after treatment were generally within the respective reference range, although some values deviated from the range slightly. However, these deviations were not clinically relevant.

**Fig 2:**
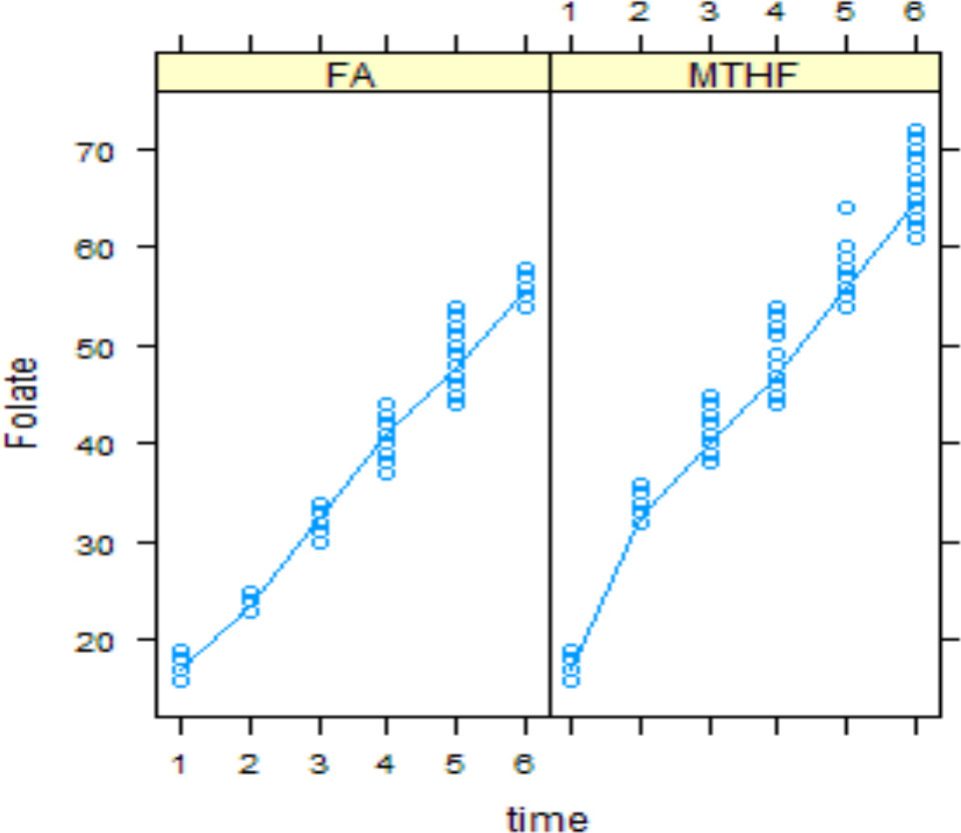
Serum folate concentration in folic acid and MTHFR groups over time.

**Fig 3:**
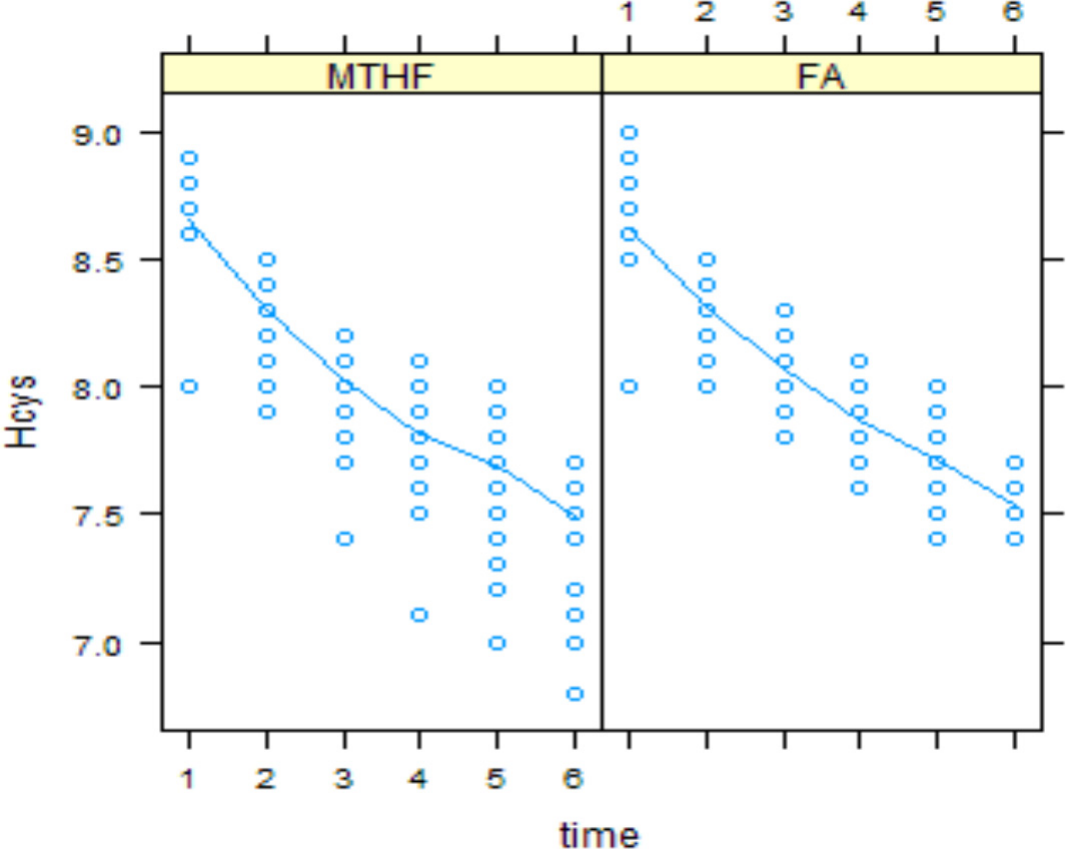
Serum tHcys concentration in folic acid and MTHFR groups over time.

Plasma folate and tHcys concentrations at each time point are shown in Figs 2 and 3, respectively. There was no significant difference between two groups in baseline values.

Serum folate increased significantly in both groups over time; these changes were significantly higher in group receiving 5-MTHF than group receiving folic acid (value = 2.39, p<0.01) and result was the same by considering the time (value = 1.24, p<0.01) (Fig 2).

Plasma tHcys decreased significantly in both groups over time; however these changes were not significantly different between the groups (value = 0.01, p = 0.147) (Fig 3).

The mixed regression model for serum folate concentration with all predictors produced (R² = 0.93). Standard deviation of random effects for intercept, time and residual was 0.0031, 0.0059 and 0.0019, respectively.

**Fig 4:**
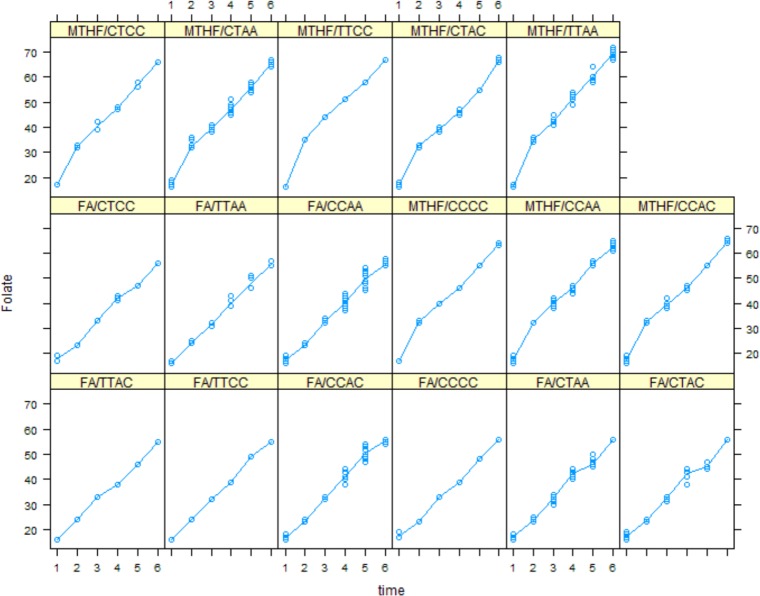
Serum folate concentration in folic acid and MTHFR groups in different MTHFR polymorphisms over time.

**Fig 5:**
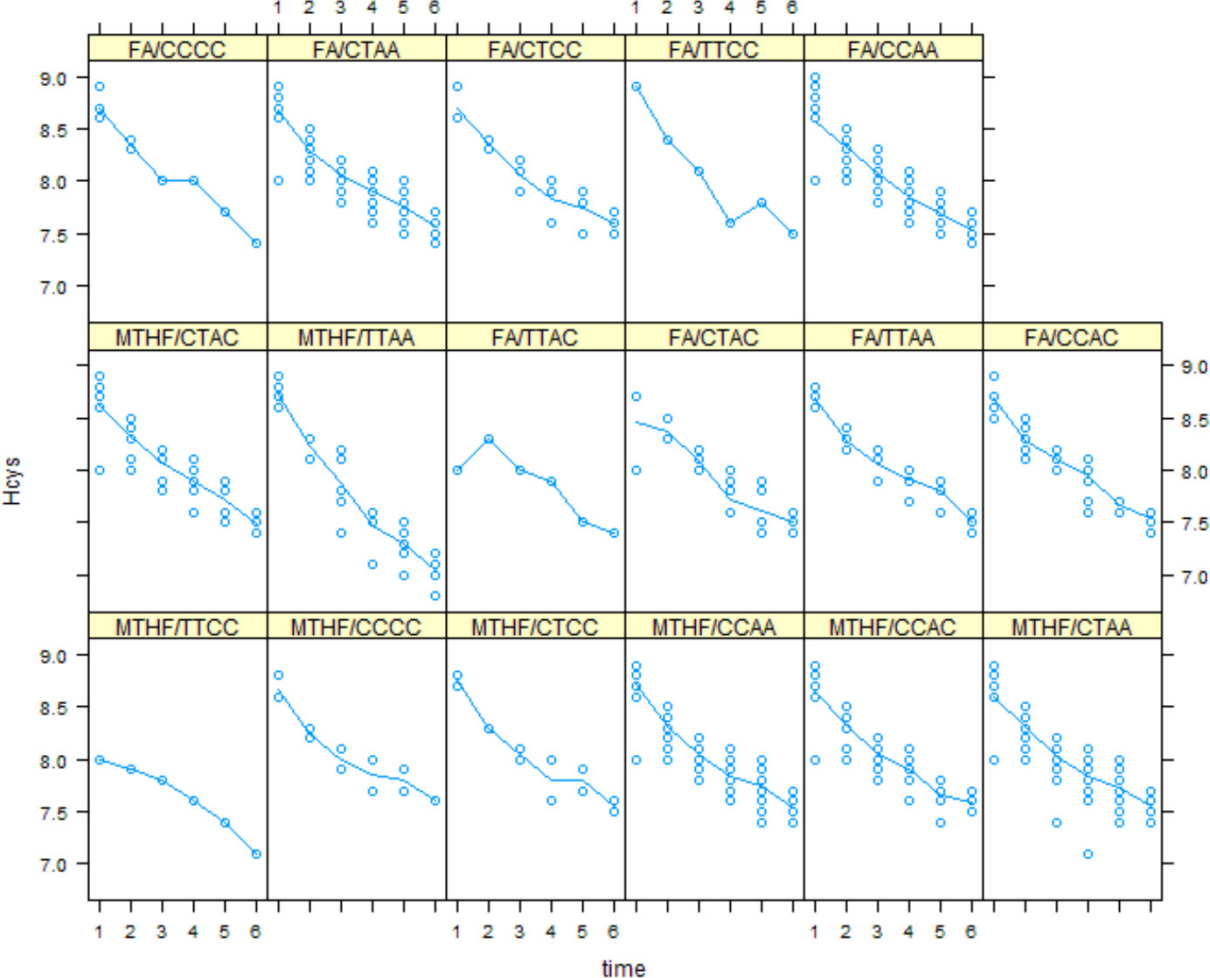
Serum tHcys concentration in folic acid and MTHFR groups in different MTHFR polymorphisms over time.

Plasma folate and tHcys concentrations with respect to MTHFR polymorphism, at each time point are shown in Figs 4 and 5, respectively. Only genotype TTAA had significant different folate value from reference group, CCAA (value = -0.98 (SE = 0.52), p = 0.05). Trend of change in serum folate over time in patients with CCAC, CTAA and TTAA genotype was significantly different from the reference group (p = .056, p = .051, p<00.1).

Only genotype TTAC had significant different tHcys value from reference group (value = -0.38 (SE = 0.19), p = 0.05). Trend of changes in serum tHcys in TTAA genotype was significantly different from reference group (p<0.01).

The mixed regression model for serum tHcys concentration with all predictors produced (R² = 0.83). Standard deviation of random effects for intercept, time and residual was 0.13, 0.03 and 0.15, respectively.

## Discussion

To our knowledge, this is the first study comparing the effects of folic acid vs. 5-MTHF in the management of recurrent abortion considering MTHFR gene polymorphism. The results of this long term double blind clinical trial have shown that the 5-MTHF, in comparison to the same amount of folic acid, could significantly increase serum folate concentration; however there was no significant difference in serum homocystein concentration and abortion rate between two groups.

There is no study comparing the effects of folic acid vs. 5-MTHF in women with recurrent abortion; however, some previous studies have compared the effect of these supplements on serum folate and homocysteine in healthy women. Our results are consistent with previous studies on healthy women which have shown that administration of 5-MTHF is more effective than folic acid supplementation at improving folate status[23], and preventing neural tube defects in early pregnancy[24, 25]. Moreover, 5-MTHF was shown to be an adequate alternative to folic acid in reducing tHcy concentrations in healthy women [26].

It seems that MTHFR polymorphism can affect the folate and MTHF supplementation efficacy; however, we found that only genotype TTAA had significant different folate value from CCAA genotype. Previous studies have shown controversial results. Fohr et al have shown that women with the TT genotype benefit the most from supplementation with either FA or MTHF [27]. Prinz-Langenohl et al have reported that 5-MTHF increases plasma folate more effectively than folic acid irrespective of the 677CT mutation of the MTHFR[28].

Recent studies suggest that MTHFR polymorphism is a genetic risk factor for pregnancy loss and neural tube defects [29, 30]; however, the results on the effect of MTHFR polymorphism in the response to tHcy-lowering therapy are controversial. A meta-analysis study results have shown a significant association between MTHFR C677T mutation and IRA, but no significance in MTHFR A1298C mutation[31]; however, Creus et al have reported that IRA is not associated with the MTHFR gene mutation[32]. Other meta-analyses supported the association between MTHFR C677T genotype and increased risk of IRA, except for Caucasians[33], and Chinese population [34].

Our data showed that 5-MTHF increased serum folate significantly more than folic acid, but the serum homocystein was not significantly different between two groups, which might be due to presence of autoantibodies that bind to folate receptors and can block the cellular uptake of folate [35]; however, Molloy et al found no association between the presence and titer of maternal folate-receptor autoantibodies and a neural-tube defect-affected pregnancy in the Irish population [36].One of the limitations of this study is that we did not measure folate-receptor autoantibodies. The other limitations are selection bias, and the concurrent therapies undertaken by the patients, which we tried to overcome these limitations by randomization assignment of the two groups. Furthermore, the optimistic sample size estimation, and the high rate of lost to follow up could be the limitations of this study; however, we tried to overcome them by the intention to treat analysis.

In conclusion, this long term, randomized double-blind, placebo-controlled study does not support any beneficial effect of 5-MTHF vs. folate supplementation in women with recurrent abortion with any MTHFR C677T and/or A1298C polymorphism except for serum folate concentration.

## Supporting Information

S1 File Study Protocol. (DOCX)

S2 File Farsi Protocol. (DOC)

S3 File Consort. (DOCX)

## References

[1] Scholl TO, Johnson WG. Folic acid: influence on the outcome of pregnancy. Am J Clin Nutr. 2000;71(5 Suppl):1295S–303S. Epub 2000/05/09. . 10799405 10.1093/ajcn/71.5.1295s

[2] Steegers-Theunissen RP, Boers GH, Blom HJ, Trijbels FJ, Eskes TK. Hyperhomocysteinaemia and recurrent spontaneous abortion or abruptio placentae. Lancet. 1992;339(8801):1122–3. Epub 1992/05/02. . 1349147 10.1016/0140-6736(92)90725-i

[3] Vollset SE, Refsum H, Irgens LM, Emblem BM, Tverdal A, Gjessing HK, et al Plasma total homocysteine, pregnancy complications, and adverse pregnancy outcomes: the Hordaland Homocysteine study. Am J Clin Nutr. 2000;71(4):962–8. Epub 2000/03/25. . 10731504 10.1093/ajcn/71.4.962

[4] Brouwer IA, van Dusseldorp M, Thomas CM, Duran M, Hautvast JG, Eskes TK, et al Low-dose folic acid supplementation decreases plasma homocysteine concentrations: a randomized trial. Am J Clin Nutr. 1999;69(1):99–104. Epub 1999/01/30. . 9925130 10.1093/ajcn/69.1.99

[5] Gindler J, Li Z, Berry RJ, Zheng J, Correa A, Sun X, et al Folic acid supplements during pregnancy and risk of miscarriage. Lancet. 2001;358(9284):796–800. Epub 2001/09/21. . 11564486 10.1016/s0140-6736(01)05969-4

[6] George L, Mills JL, Johansson AL, Nordmark A, Olander B, Granath F, et al Plasma folate levels and risk of spontaneous abortion. JAMA. 2002;288(15):1867–73. Epub 2002/10/17. doi: joc20985 [pii]. . 12377085 10.1001/jama.288.15.1867

[7] Bergen NE, Jaddoe VW, Timmermans S, Hofman A, Lindemans J, Russcher H, et al Homocysteine and folate concentrations in early pregnancy and the risk of adverse pregnancy outcomes: the Generation R Study. BJOG: an international journal of obstetrics and gynaecology. 2012;119(6):739–51. Epub 2012/04/12. 10.1111/j.1471-0528.2012.03321.x . 22489763

[8] Byrne J. Periconceptional folic acid prevents miscarriage in Irish families with neural tube defects. Irish journal of medical science. 2011;180(1):59–62. Epub 2010/11/06. 10.1007/s11845-010-0629-5 . 21052862

[9] Wouters MG, Boers GH, Blom HJ, Trijbels FJ, Thomas CM, Borm GF, et al Hyperhomocysteinemia: a risk factor in women with unexplained recurrent early pregnancy loss. Fertil Steril. 1993;60(5):820–5. Epub 1993/11/01. . 8224267

[10] Nelen WL, Blom HJ, Steegers EA, den Heijer M, Thomas CM, Eskes TK. Homocysteine and folate levels as risk factors for recurrent early pregnancy loss. Obstet Gynecol. 2000;95(4):519–24. Epub 2000/03/22. . 10725483 10.1016/s0029-7844(99)00610-9

[11] Ronnenberg AG, Goldman MB, Chen D, Aitken IW, Willett WC, Selhub J, et al Preconception folate and vitamin B(6) status and clinical spontaneous abortion in Chinese women. Obstet Gynecol. 2002;100(1):107–13. Epub 2002/07/09. . 12100811 10.1016/s0029-7844(02)01978-6

[12] Yamada T, Morikawa M, Yamada T, Kishi R, Sengoku K, Endo T, et al First-trimester serum folate levels and subsequent risk of abortion and preterm birth among Japanese women with singleton pregnancies. Archives of gynecology and obstetrics. 2013;287(1):9–14. Epub 2012/08/10. 10.1007/s00404-012-2501-5 . 22875049

[13] Zhang X, Li J, Gu Y, Zhao Y, Wang Z, Jia G. A pilot study on environmental and behavioral factors related to missed abortion. Environmental health and preventive medicine. 2011;16(4):273–8. Epub 2011/03/25. 10.1007/s12199-010-0196-4 ; PubMed Central PMCID: PMCPmc3117210. 21431815 PMC3117210

[14] Charles DH, Ness AR, Campbell D, Smith GD, Whitley E, Hall MH. Folic acid supplements in pregnancy and birth outcome: re-analysis of a large randomised controlled trial and update of Cochrane review. Paediatric and perinatal epidemiology. 2005;19(2):112–24. Epub 2005/03/25. 10.1111/j.1365-3016.2005.00633.x . 15787886

[15] Chiaffarino F, Ascone GB, Bortolus R, Mastroia-Covo P, Ricci E, Cipriani S, et al [Effects of folic acid supplementation on pregnancy outcomes: a review of randomized clinical trials]. Minerva ginecologica. 2010;62(4):293–301. Epub 2010/09/10. . 20827247

[16] Furness D, Fenech M, Dekker G, Khong TY, Roberts C, Hague W. Folate, vitamin B12, vitamin B6 and homocysteine: impact on pregnancy outcome. Maternal & child nutrition. 2013;9(2):155–66. Epub 2011/10/26. 10.1111/j.1740-8709.2011.00364.x . 22023381 PMC6860531

[17] Kim MW, Ahn KH, Ryu KJ, Hong SC, Lee JS, Nava-Ocampo AA, et al Preventive effects of folic acid supplementation on adverse maternal and fetal outcomes. PLoS One. 2014;9(5):e97273 Epub 2014/05/21. 10.1371/journal.pone.0097273 ; PubMed Central PMCID: PMCPmc4026223. 24842467 PMC4026223

[18] Shi H, Yang S, Liu Y, Huang P, Lin N, Sun X, et al Study on Environmental Causes and SNPs of MTHFR, MS and CBS Genes Related to Congenital Heart Disease. PLoS One. 2015;10(6):e0128646 Epub 2015/06/04. 10.1371/journal.pone.0128646 PONE-D-14-52335 [pii]. 26035828 PMC4452709

[19] Nelen WL, Blom HJ, Thomas CM, Steegers EA, Boers GH, Eskes TK. Methylenetetrahydrofolate reductase polymorphism affects the change in homocysteine and folate concentrations resulting from low dose folic acid supplementation in women with unexplained recurrent miscarriages. J Nutr. 1998;128(8):1336–41. Epub 1998/08/04. . 9687553 10.1093/jn/128.8.1336

[20] Tempfer CB, Kurz C, Bentz EK, Unfried G, Walch K, Czizek U, et al A combination treatment of prednisone, aspirin, folate, and progesterone in women with idiopathic recurrent miscarriage: a matched-pair study. Fertil Steril. 2006;86(1):145–8. Epub 2006/05/24. 10.1016/j.fertnstert.2005.12.035 . 16716321

[21] Quere I, Mercier E, Bellet H, Janbon C, Mares P, Gris JC. Vitamin supplementation and pregnancy outcome in women with recurrent early pregnancy loss and hyperhomocysteinemia. Fertil Steril. 2001;75(4):823–5. Epub 2001/04/05. . 11287044 10.1016/s0015-0282(01)01678-8

[22] Zetterberg H, Zafiropoulos A, Spandidos DA, Rymo L, Blennow K. Gene-gene interaction between fetal MTHFR 677C>T and transcobalamin 776C>G polymorphisms in human spontaneous abortion. Hum Reprod. 2003;18(9):1948–50. Epub 2003/08/19. . 12923155 10.1093/humrep/deg375

[23] Lamers Y, Prinz-Langenohl R, Bramswig S, Pietrzik K. Red blood cell folate concentrations increase more after supplementation with [6S]-5-methyltetrahydrofolate than with folic acid in women of childbearing age. Am J Clin Nutr. 2006;84(1):156–61. Epub 2006/07/11. doi: 84/1/156 [pii]. . 16825690 10.1093/ajcn/84.1.156

[24] Obeid R, Holzgreve W, Pietrzik K. Is 5-methyltetrahydrofolate an alternative to folic acid for the prevention of neural tube defects? J Perinat Med. 2013;41(5):469–83. Epub 2013/03/14. 10.1515/jpm-2012-0256 /j/jpme.ahead-of-print/jpm-2012-0256/jpm-2012-0256.xml [pii]. . 23482308

[25] Ray JG, Laskin CA. Folic acid and homocyst(e)ine metabolic defects and the risk of placental abruption, pre-eclampsia and spontaneous pregnancy loss: A systematic review. Placenta. 1999;20(7):519–29. Epub 1999/08/24. 10.1053/plac.1999.0417 . 10452905

[26] Lamers Y, Prinz-Langenohl R, Moser R, Pietrzik K. Supplementation with [6S]-5-methyltetrahydrofolate or folic acid equally reduces plasma total homocysteine concentrations in healthy women. Am J Clin Nutr. 2004;79(3):473–8. Epub 2004/02/27. . 14985224 10.1093/ajcn/79.3.473

[27] Fohr IP, Prinz-Langenohl R, Bronstrup A, Bohlmann AM, Nau H, Berthold HK, et al 5,10-Methylenetetrahydrofolate reductase genotype determines the plasma homocysteine-lowering effect of supplementation with 5-methyltetrahydrofolate or folic acid in healthy young women. Am J Clin Nutr. 2002;75(2):275–82. Epub 2002/01/30. . 11815318 10.1093/ajcn/75.2.275

[28] Prinz-Langenohl R, Bramswig S, Tobolski O, Smulders YM, Smith DE, Finglas PM, et al [6S]-5-methyltetrahydrofolate increases plasma folate more effectively than folic acid in women with the homozygous or wild-type 677C—>T polymorphism of methylenetetrahydrofolate reductase. Br J Pharmacol. 2009;158(8):2014–21. Epub 2009/11/18. doi: BPH492 [pii] 10.1111/j.1476-5381.2009.00492.x 19917061 PMC2807663

[29] Kirke PN, Mills JL, Molloy AM, Brody LC, O'Leary VB, Daly L, et al Impact of the MTHFR C677T polymorphism on risk of neural tube defects: case-control study. BMJ. 2004;328(7455):1535–6. Epub 2004/05/25. 10.1136/bmj.38036.646030.EE bmj.38036.646030.EE [pii]. 15155469 PMC437144

[30] Nair RR, Khanna A, Singh R, Singh K. Association of maternal and fetal MTHFR A1298C polymorphism with the risk of pregnancy loss: a study of an Indian population and a meta-analysis. Fertil Steril. 2013;99(5):1311–8 e4. Epub 2013/01/30. doi: S0015-0282(12)02536-8 [pii] 10.1016/j.fertnstert.2012.12.027 . 23357458

[31] Cao Y, Xu J, Zhang Z, Huang X, Zhang A, Wang J, et al Association study between methylenetetrahydrofolate reductase polymorphisms and unexplained recurrent pregnancy loss: a meta-analysis. Gene. 2013;514(2):105–11. Epub 2012/12/04. doi: S0378-1119(12)01396-0 [pii] 10.1016/j.gene.2012.10.091 . 23201418

[32] Creus M, Deulofeu R, Penarrubia J, Carmona F, Balasch J. Plasma homocysteine and vitamin B12 serum levels, red blood cell folate concentrations, C677T methylenetetrahydrofolate reductase gene mutation and risk of recurrent miscarriage: a case-control study in Spain. Clin Chem Lab Med. 2013;51(3):693–9. Epub 2012/10/26. 10.1515/cclm-2012-0452 /j/cclm.ahead-of-print/cclm-2012-0452/cclm-2012-0452.xml [pii]. . 23095199

[33] Wu X, Zhao L, Zhu H, He D, Tang W, Luo Y. Association between the MTHFR C677T polymorphism and recurrent pregnancy loss: a meta-analysis. Genet Test Mol Biomarkers. 2012;16(7):806–11. Epub 2012/02/09. 10.1089/gtmb.2011.0318 . 22313097

[34] Ren A, Wang J. Methylenetetrahydrofolate reductase C677T polymorphism and the risk of unexplained recurrent pregnancy loss: a meta-analysis. Fertil Steril. 2006;86(6):1716–22. Epub 2006/11/01. doi: S0015-0282(06)03070-6 [pii] 10.1016/j.fertnstert.2006.05.052 . 17074326

[35] Rothenberg SP, da Costa MP, Sequeira JM, Cracco J, Roberts JL, Weedon J, et al Autoantibodies against folate receptors in women with a pregnancy complicated by a neural-tube defect. N Engl J Med. 2004;350(2):134–42. Epub 2004/01/09. 10.1056/NEJMoa031145350/2/134 [pii]. . 14711912

[36] Molloy AM, Quadros EV, Sequeira JM, Troendle JF, Scott JM, Kirke PN, et al Lack of association between folate-receptor autoantibodies and neural-tube defects. N Engl J Med. 2009;361(2):152–60. Epub 2009/07/10. doi: 361/2/152 [pii] 10.1056/NEJMoa0803783 19587340 PMC4149290

