# Supplementary material for: Methyltetrahydrofolate vs Folic Acid Supplementation in Idiopathic Recurrent Miscarriage with Respect to Methylenetetrahydrofolate Reductase C677T and A1298C Polymorphisms: A Randomized Controlled Trial
Source: PLoS One. 2015 Dec 2;10(12):e0143569. doi: 10.1371/journal.pone.0143569 (PMC4668025; doi:10.1371/journal.pone.0143569)
Supplement: S1 File — (DOCX) [file pone.0143569.s001.docx]

**Study Protocol**

**Study Design and Participants**

The patients will be recruited from April 2011 from Recurrent Abortion clinics in Tehran, Iran. Eligible patients will be informed about the study procedures by their gynecologist or trained clinic staffs. After a full review of the inclusion and exclusion criteria and explanation of the risks and benefits of the study, women with three or more idiopathic abortion who fill out the written consent form to participate, will be enrolled. All women will take a standard diagnostic workup to rule out the presence of antiphospholipid syndrome or anatomic, cytogenetic, hormonal, or infectious pathologies. Diagnostic procedures include hysteroscopy; paternal and maternal karyotype; cervical cultures; a comprehensive hormonal status, and evaluation of antiphospholipid syndrome with IgM and IgG anticardiolipin antibody assessment and lupus anticoagulant testing.

Participants’ self-described age, race ethnicity, demographic information, medical, occupational and family histories, lifetime history of tobacco use, intake of nutritional supplements, lifestyle and environmental exposures will be recorded for preplanned analyses of possible moderators of the primary outcome.

**Randomization, Masking and Interventions**

Randomization should be stratified by age and the number of abortions. Participants will be assigned using computer-generated randomization to receive either 1mg folic acid or 1 mg 5-MTHF during the study period. The group assignments will be concealed in sealed envelopes and opened at enrollment by somebody who is blinded to all baseline assessments.

**Treatment and Follow-up**

The participants will be evaluated by their Gynecologist at baseline, after 8 weeks, and at the gestational age of 4, 8, 12, and 20 weeks; women who will not get pregnant within six months of the study beginning, will be excluded from the study. Study protocol adherence and plasma folate and homocystein concentration will be assessed at all of these visits while plasma vitamin B_12_, Pyridoxal phosphate (PLP), and MTHFR gene polymorphisms for C677T and A1298C will be evaluated at randomization time. Weekly telephone contacts will be made to encourage adherence, and answer study-related questions. Adverse effects, adverse events, and medical status will be recorded at each contact. The supplements will be delivered to the patients every four weeks.

**Primary and Secondary Outcomes**

The ongoing pregnancy at week 20^th^ is the primary outcome measure. Secondary outcomes include serum folate and tHcy concentration at the beginning, after 8 weeks, and at the gestational age of 4, 8, 12, and 20 week.

**Procedures**

Information on baseline characteristics will be obtained by interviewer administered questionnaire at the first visit and included data on demographics, occupation, medical history, number of previous abortions, smoking, and current medications. Folate, tHcy, Vitamin B_12,_ and PLP concentrations in EDTA-treated plasma will be measured in EDTA-treated plasma using their commercially available immunoassay kits.

MTHFR polymorphism will be assessed using PCR-RFLP.
