# Supplementary material for: Methyltetrahydrofolate vs Folic Acid Supplementation in Idiopathic Recurrent Miscarriage with Respect to Methylenetetrahydrofolate Reductase C677T and A1298C Polymorphisms: A Randomized Controlled Trial
Source: PLoS One. 2015 Dec 2;10(12):e0143569. doi: 10.1371/journal.pone.0143569 (PMC4668025; doi:10.1371/journal.pone.0143569)
Supplement: S2 File — (DOC) [file pone.0143569.s002.doc]

بسمه تعالي


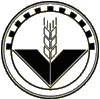


انستيتو تحقيقات تغذيه‌اي و صنايع غذايي كشور

معاونت پژوهشي

كاربرگ پيشنهاد طرح پژوهشي

فرم شماره 2

1- مشخصات كلي طرح

|  | | عنوان طرح: | | | | مقایسه تاثیر اسید فولیک و 5 متیل تترا هیدروفولات بر سطح سرمی فولات، هموسیستئین و میزان سقط در زنان مبتلا به سقط مکرر | | | | | | | |
| --- | --- | --- | --- | --- | --- | --- | --- | --- | --- | --- | --- | --- | --- |
| عنوان طرح (انگليسي): | | | | Comparison of the effect of folic acid and 5-methyltetrahydrofolate (5MTHF) on serum folat and homocysteine levels, and abortion rates in women suffering from recurrent abortion | | | | | | | | | |
| نام مجري/مجريان طرح: | | | | | دكتر آزیتا حکمت دوست، دکتر سهیلا عارفی، آقای فرهاد وحید | | | | | | | | |
|  | تاريخ تقريبي شروع طرح: | | | | | | تابستان 89 | | | | | | |
| طول مدت اجراي طرح: | | | | | 18 | | | ماه | | | | | |
|  | | | *كل بودجه مورد نياز انجام طرح تحقيقاتي: | | | | | | | 69950000 | | | ريال |
|  | | | مجموع نيروي انساني: | | | | | | | 0 | | | ريال |
|  | | | جمع هزينه هاي آزمايشات و خدمات تخصصي*:* | | | | | | |  | | | ريال |
|  | | | جمع هزينه هاي وسايل و مواد مصرفي: | | | | | | | 69950000 | | | ريال |
|  | | | جمع هزينه هاي وسايل و مواد غيرمصرفي: | | | | | | |  | | | ريال |
|  | | | جمع هزينه هاي مسافرت: | | | | | | |  | | | ريال |
|  | | | جمع هزينه‌هاي تكثير و تايپ: | | | | | | |  | | | ريال |
|  | | | ساير هزينه ها: | | | | | | |  | | | ريال |
| *بودجه‌اي كه از طرف انستيتو در مجموع جهت اجراي طرح پرداخت خواهد شد. | | | | | | | | | | |  |  | |
| نوع طرح: | | | | | پايه‌اي | | | | كاربردي-پايه‌اي | | | *كاربردي | |
| پايان نامه دانشجويي: | | | | | مقطع فوق ليسانس٭ | | | | مقطع دكترا | | | | |
| در صورت مشترك بودن طرح، نام سازمان/سازمانهاي همكار ذكر شود. پژوهشگاه ابن سینا | | | | | | | | | | | | | |
| كل بودجه اجراي طرح: 69950000  مقدار بودجه‌اي كه انستيتو تقبل مي‌نمايد.34975000  مقدار بودجه‌اي كه سازمان همكار جهت انجام خدمات مربوط تقبل مي‌نمايد34975000 | | | | | | | | | | | | | |

*2- نكات مهم*

مجري مسئول فردي از گروه تحقيق است كه در قرارداد مشخص مي‌شود و مسئوليت كليه مراحل اجرائي، قانوني و حقوقي طرح را بر عهده داشته و تمامي مكاتبات اداري و مالي طرح با ايشان انجام مي شود.

آدرس نويسنده اول يا نويسنده مسئول در مقاله حاصل از طرح پژوهشي در مقاله‌‌هاي فارسي و انگليسي بايد به شكل زير باشد.

آدرس فارسي: (ذكر گروه) انستيتو تحقيقات تغذيه‌اي و صنايع غذايي كشور، دانشكده علوم تغذيه و صنايع غذايي، دانشگاه علوم پزشكي شهيد بهشتي

آدرس انگليسي: (Department) National Nutrition and Food Technology Research Institute, Faculty of Nutrition Sciences and Food Technology, Shahid Beheshti University of Medical Sciences, Tehran, Iran

يك نسخه از مقاله حاصل از طرح پس از submission جهت كنترل موارد مورد نظر انستيتو به معاونت پژوهشي تحويل گردد.

يك نسخه از مقاله پس از چاپ جهت حفظ در سوابق به معاونت پژوهشي ارائه گردد (حتي پس ازاتمام قرارداد و تسويه حساب نهايي).

بر اساس دستورالعمل وزارت بهداشت چنانچه طرح تحقيقاتي، پايان نامه نيز باشد، در متن مقاله حاصله اين موضوع تصريح شود.

جمله زير در مقاله حتما" بايد ذكر شود:

"اين مقاله حاصل طرح پژوهشي ........(نام طرح) مي‌باشد كه انستيتو تحقيقات تغذيه‌اي و صنايع غذايي كشور بودجه اجراي آن را تآمين كرده است."

ثبت كارآزمائي باليني در سايت <http://www.irct.ir/fa> قبل از درخواست پيش پرداخت اول انجام شود.

پرداخت حداكثر 25 درصد از حق التحقيق طرح به عنوان حسن انجام كار منوط به احراز شرايط زير مي باشد:

الف –اتمام به موقع طرح : هر يك از طرح هاي تحقيقاتي مجاز به يكبار تمديد قرارداد مي باشند. بيش از آن به ازاي هر بار تمديد مهلت قرارداد ده درصد از ميزان حسن انجام كار كسر خواهد شد.

ب – نمره گزارش نهائي: در صورت كسب نمره متوسط 5 درصد و نمره ضعيف 10 درصد از ميزان حسن انجام كار كسر خواهد شد.

*امضاء تمام مجريان:*

*مجري مسئول: ..........................................*

*مجري دوم: ................................................*

اسامي و مشخصات گروه پژوهش

عنوان طرح: مقایسه تاثیر اسید فولیک و 5 متیل تترا هیدروفولات بر سطح سرمی فولات، هموسیستئین و میزان سقط در زنان مبتلا به سقط مکرر

مجريان طرح

| رديف | نام و نام خانوادگي | نشاني الكترونيكي  (E-mail) | تلفن همراه | رشته/گرايش تخصصي | امضاء |
| --- | --- | --- | --- | --- | --- |
|  | دکتر آزیتا حکمت دوست | a_hekmat2000@yahoo.com | 09123065084 | تغذیه |  |
|  | دکتر سهیلا عارفی | sarefi@yahoo.com | 09121157889 | زنان و زایمان (ناباروری) |  |
|  | فرهاد وحید | ostadvahid@gmail.com | 09370684415 | تغذیه |  |

همكاران اصلي طرح

| رديف | نام و نام خانوادگي | نشاني الكترونيكي  (E-mail) | تلفن همراه | رشته/گرايش تخصصي | امضاء |
| --- | --- | --- | --- | --- | --- |
|  | نیکنام لک پور |  |  |  |  |
|  |  |  |  |  |  |
|  |  |  |  |  |  |

مشاوران طرح

| رديف | نام و نام خانوادگي | نشاني الكترونيكي  (E-mail) | تلفن همراه | رشته/گرايش تخصصي | امضاء |
| --- | --- | --- | --- | --- | --- |
|  | دکتر بهرام رشیدخانی |  |  | اپیدمیولوژی |  |
|  | دکتر محمدرضا صادقی |  |  | بیوشیمی |  |

** در صورتي كه محل كار مجري خارج از انستيتو و دانشكده باشد, كارنامه فعاليتهاي پژوهشي (CV) ضميمه گردد.*

4- چكيده طرح پيشنهادي

کمبود فولات شایع ترین کمبود مشاهده شده در زنان پس از آهن بوده و بروز آن در بارداری تقریبا 30% است. سقط های مکرر برای خانواده و تیم درمانی آنها معضل بسیار بزرگی محسوب می شود. سقط خود به خودی (Spontaneous Abortion) تقریبا در 40 درصد بارداری ها رخ می دهد. غلظت های پایین اسید فولیک نیز با سقط مکرر مرتبط است. مطالعات اپیدمیولوژیک موید نقش هموسیستئین در عوارض بارداری از جمله قطع جفت (Placental Abortion)، پره اکلامپسی و سقط خود به خود می باشد. علاوه بر این، هموسیستئین پلاسما یک نشانگر حساس وضعیت فولات محسوب می شود. هیپرهموسیستئینمی (Hyper Homocysteinemia) (HHcy) و جهش هموزیگوت C677T در ژن 10-5 متیلن تترا هیدروفولات ردوکتاز (Metylen Tetra Hydro Folat Reductase) (MTHFR) با افزایش خطر سقط بارداری مکرر (Recurrent Pregnancy Loss) مرتبط است.

چندین مطالعه بر روی رابطه ویتامین های گروه B به خصوص فولات با سقط خود به خودی منتشر شده است ولی داده های موجود در مورد سقط مکرر محدود است. در مطالعات پیشین نشان داده شد که مکمل یاری روزانه اسید فولیک در زنان دارای سابقه سقط مکرر بدون علت (unexplained) موجب کاهش قابل ملاحظه هموسیستئین شد. همچنین درمان ترکیبی با پردنیزون، آسپرین، فولات و پروژسترون با میزان تولد زنده بالاتر در مقایسه با عدم درمان در زنان دارای سابقه سقط مکرر بدون علت Idiopathic (Idiopathic Recurrent Miscarriages) (IRM) مرتبط بود.

شایع ترین درمان به کار رفته برای سقط جنین مکرر تجویز اسید فولیک بوده و در زنان با سابقه ی سقط بارداری زود هنگام (Pregnancy Loss) مفید می باشد و به نظر می رسد که درمان انتخابی برای کاهش غلظت هموسیستئین تام پلاسما است. بنابراین تجویز دوزهای درمانی فولات در زنان دارای IRM منطقی به نظر می رسد. مزیت احتمال (L-Metyl Tetra Hydro Folat) L-MTHF نسبت به اسید فولیک این است که احتمال پنهان ماندن علایم هماتولوژیک کمبود ویتامین B12 حتی با دریافت بالای L-MTHF نیز بعید می باشد. دوز پایین L-5MTHF در کاهش غلظت هموسیستئین در اشخاص سالم، موثرتر از اسید فولیک می باشد. با توجه به اثرات مشابه این دو فرم بر روی عملکرد عروقی و نیز کاهش (total Homocysteine) tHcy به نظر می رسد که هرگونه مزیت بالینی مرتبط با اسید فولیک با استفاده از L-MTHF نیز قابل دستیابی است.

از آنجاییکه تا کنون مطالعه ای در زمینه ی اثرات مکمل یاری L-5MTHF بر روی زنان مبتلا به سقط مکرر صورت نگرفته است، این مطالعه با هدف مقایسه تاثیر اسید فولیک و 5 متیل تترا هیدروفولات بر سطح سرمی فولات، هموسیستئین و میزان سقط در زنان مبتلا به سقط مکرر انجام می شود. امید است نتایج این مطالعه در ارائه درمان موثرتر و کم عارضه برای زنان مبتلا به سقط مکرر موثر باشد.

**اهداف مطالعه**

**هدف کلی :**

مقایسه تاثیر اسید فولیک و 5 متیل تترا هیدروفولات بر سطح سرمی فولات، هموسیستئین و میزان سقط در زنان مبتلا به سقط مکرر

**اهداف اختصاصی :**

- تعیین متغیرهای سن، وزن، قد، نمایه توده بدن، و تعداد سقط مکرر قبل از آغاز مطالعه، وضعیت سقط زنان مورد مطالعه در دو گروه در زمان شروع مطالعه
- تعیین ژنوتیپ C677T ژن MTHFR، و ژنوتیپ A1298C ژن MTHFR در دو گروه در زمان شروع مطالعه
- تعیین متغیرهای تعداد حاملگی بیشتر از 20 هفته پس از آغاز مطالعه، تعداد سقط کمتر از 20 هفته پس از آغاز مطالعه، تعداد هفته بارداری قبل از زایمان یا سقط در زنان مبتلا به سقط مکرر در دو گروه مورد مطالعه
- تعیین متغیرهای هموسیستئین پلاسما، و فولات پلاسما در زمان شروع و هفته هشتم مطالعه و نیز هفته ششم، دوازدهم و بیستم حاملگی در دو گروه مورد مطالعه.
- مقایسه ميانگين تعداد حاملگی بیشتر از 20 هفته پس از آغاز مطالعه، تعداد سقط کمتر از 20 هفته پس از آغاز مطالعه، تعداد هفته بارداری قبل از زایمان یا سقط در پایان مطالعه بین دو گروه مورد مطالعه.
- مقایسه میانگین متغیرهای هموسیستئین پلاسما و فولات پلاسما در زمان شروع و هفته هشتم مطالعه و نيز هفته ششم، دوازدهم و بیستم حاملگی بین دو گروه مورد مطالعه و در هر گروه بین زمان های مطالعه.
- مقایسه میانگین تغییرات هموسیستئین پلاسما و فولات پلاسما در طول مدت مطالعه بین دو گروه مورد بررسی.

**نوع مطالعه:**

مطالعه حاضر یک کارآزمایی بالینی تصادفی (Randomized Clinical Trial) می باشد.

**نمونه مورد مطالعه:**

در این تحقیق نمونه های مورد مطالعه از میان زنان مبتلا به سقط مکرر مراجعه کننده به مركز فوق‌تخصصی درمان ناباروری و سقط مكرر ابن‌سينا مطابق با معیار های زیر انتخاب می شوند:

**الف- معیارهای ورود به مطالعه**

- تمایل به همکاری و تکمیل فرم رضایت نامه آگاهانه.

- حداقل 6 ماه از آخرین سقط آنها گذشته باشد.

- حداقل سابقه سه یا بیش از سه سقط جنین متوالی خود به خودی از یک همسر داشته باشند.

- عدم دریافت مکمل های حاوی اسيد فوليك در 6 ماه اخیر یا دریافت بالا از غذاهای غنی شده با فولات.

- سقط های افراد فاقد پاتولوژی آناتومیک، سیتولوژیک، هورمونی، عفونی یا سندرم آنتی فسفولیپید باشد.

- عدم داشتن رژیم غذایی خاص از جمله گیاهخواری.

**ب- معیارهای خروج از مطالعه**

- سقط های عمدی یا انتخابی (elective)، و حاملگی های نابجا (ectopic).

- وجود بدخیمی های فعال، ناهنجاری های کروموزمی، آنومالی های شدید رحمی، اختلال عملکرد تیروئید، اختلال عملکرد کلیوی یا کبدی، عدم تحمل گلوکز، صرع، اختلالات اندوکرین، ایمونولوژیک، صفراوی و گوارشی، مصرف الکل یا مواد مخدر.

- مصرف قرص های ضدبارداری خوراکی یا داروهایی که بر متابولیسم متیونین- هموسیستئین یا فولات اثر می گذارند.

- تغییر در پس زمینه دارویی.

**حجم نمونه و روش نمونه گیری :**

در این مطالعه تعداد نمونه برای هر یک از گروهها 30 نفر در نظر گرفته شد. نمونه ها از مركز فوق ‌تخصصی درمان ناباروری و سقط مكرر ابن‌سينا با روش نمونه گیری آسان (Convenience Sampling) انتخاب می گردند.

**روش اجرای مطالعه**

در این مطالعه ابتدا از زنان مبتلا به سقط مکرر مراجعه کننده به مرکز فوق‌ تخصصی درمان ناباروری و سقط مكرر ابن‌سينا که دارای معیارهای ورود به مطالعه می باشند، در صورت تمایل به شرکت در این مطالعه رضایتنامه آگاهانه کتبی از خودشان و همسرانشان اخذ می گردد. سپس زنان مورد مطالعه به طور تصادفی به 2 گروه دریافت کننده مکمل اسید فولیک و يا مکمل 5 متیل تتراهیدروفولات تقسیم می شوند. زنان در گروه دریافت کننده مکمل اسید فولیک روزانه 5 میلی گرم (یک قرص) مکمل اسید فولیک و زنان گروه دریافت کننده مکمل 5 متیل تتراهیدروفولات روزانه 5 میلی گرم (یک قرص) مکمل 5 متیل تتراهیدروفولات در طول این مطالعه 7 ماهه دریافت خواهند کرد. در شروع و هفته هشتم مطالعه و نیز هفته ششم، دوازدهم و بیستم حاملگی از تمامی زنان در حالت ناشتا 10 سی سی خون وریدی گرفته می شود و غلظت هموسیستئین پلاسما و فولات پلاسما به ترتیب با روش (Enzyme-Linked Immono Sorbent Assay) RIA (Radio Immuno Assay) ,ELISA اندازه گیری می شود. در پایان هفته هشتم مطالعه کلیه زنان دوباره ویزیت شده و از آنها خواسته می شود تا اقدام به حاملگی نمایند. همچنین در این تحقیق شاخص هاي آنتروپومتريك در شروع و هفته هشتم مطالعه و نیز هفته ششم، دوازدهم و بیستم حاملگی اندازه گیری می شود. اطلاعات مورد نیاز در مورد سن، استعمال دخانیات، تعداد سقط مکرر قبل از آغاز مطالعه، وضعیت سقط زنان مورد مطالعه در زمان شروع مطالعه با تکمیل پرسشنامه اطلاعات عمومی با مصاحبه حضوری و بررسی پرونده های پزشکی بدست می آید. همچنین فولات دریافتی از رژیم غذایی با استفاده از پرسشنامه یادآمد خوراک در مورد 3 روز هفته با مصاحبه حضوری در آغاز مطالعه و هفته هشتم و نیز هفته ششم، دوازدهم و بیستم حاملگی ارزیابی می شود. آنگاه اطلاعات بدست آمده از طریق این پرسشنامه ها با استفاده از نرم افزار تغذیه ای Nutritionist 4 مورد تجزیه و تحلیل قرار می گیرد. تعیین ژنوتیپ C677T ژن MTHFR و ژنوتیپ A1298C ژن MTHFR نیز در شروع مطالعه با روش (Polymerase Chain Reaction, Restriction Fragment Length Polymorphism) PCR,RFLP انجام می شود. همچنین در پایان این مطالعه ميانگين تعداد حاملگی بیشتر از 20 هفته پس از آغاز مطالعه، تعداد سقط کمتر از 20 هفته پس از آغاز مطالعه، تعداد هفته بارداری قبل از زایمان یا سقط بین دو گروه مورد مطالعه مقایسه می شود. در نهايت نتايج بدست آمده با استفاده از آزمون آماري آنالیز واریانس برای داده های تکراری مورد تجزيه و تحليل قرار مي گيرد.

**دستاوردهای منتظره طرح و مشکلات احتمالی**

به نظر می رسد که مکمل 5 متیل تترا هیدروفولات نسبت به مکمل اسید فولیک، در درمان سقط مکرر موثرتر باشد، ولیکن، هم اکنون از مکمل اسید فولیک برای درمان سقط مکرر استفاده می شود، اگر نتایج این مطالعه و سایر مطالعات تکمیل کننده نشان دهد که تاثیر 5 متیل تترا هیدروفولات به طور چشمگیری بیشتر از اسیدفولیک می باشد، می توان از آن به جای اسید فولیک در درمان سقط مکرر استفاده نمود.

***5- تعريف واژه ها***

*سقط مکرر: 3 یا بیش از 3 سقط جنین متوالی با اتیولوژی ناشناخته*

*مکمل اسید فولیک: 5 میلیگرم* (11325 نانو مول) *اسیدفولیک صناعی خوراکی*

*مکمل 5 متیل تتراهیدرو فولات: مکمل حاوی 5 میلی گرم* (11325 نانو مول)  *فرم متیله اسید فولیک*

*ژن MTHFR : ژن کد کننده آنزیم متیل تترا هیدروفولات ردوکتاز که دو پلی مرفیسم شایع آن* C677T *و* A1298C *می باشند.*

*سقط های عمدی یا انتخابی (elective*): سقطی که به طور عمدی و با مداخله شخص به وجود آمده باشد.

*حاملگی های نابجا (ectopic):* حاملگی که جنین در محلی غیر از داخل رحم جایگزین شده باشد.

***6- بيان مسئله تحقيق***

کمبود فولات شایع ترین کمبود مشاهده شده در زنان پس از آهن بوده و بروز آن در بارداری تقریبا 30% است (1). در بسیاری از کشورها به زنانی که قصد بارداری دارند مکمل یاری با 400 میکرو گرم اسید فولیک در روز برای کاهش خطر تولد نوزادی با نقص لوله ی عصبی (Neural Tube Defects) (NTD) توصیه می شود (2). اسید فولیک یک فرم اکسید شده سنتتیک فولات است که در مکمل ها به کار رفته و به علت پایداری و زیست دسترسی بالا به غذاها افزوده می شود. یکی از فرم های احیا شده فولات که ال- 5 متیل تتراهیدروفولات (L-5MTHF) نام دارد، به شکل مکمل از پایداری بالایی برخورداربوده (3) و مشتق سنتتیک کریستالیزه خالصی از فرم غالب فولات در طبیعت می باشد (4). سقط های مکرر (Recurrent Abortion) برای خانواده و تیم درمانی آنها معضل بسیار بزرگی محسوب می شود. سقط به صورت سقط بارداری (Pregnancy Loss) با وزن 500 گرم یا کمتر و سقط مکرر یا سقط عادتی (Habitual Abortion) به صورت سه سقط یا بیشتراز یک همسر تعریف می شود ( 5). سقط خود به خودی (Spontaneous Abortion) تقریبا در 40 درصد بارداری ها رخ می دهد (6). مکانیسم های چندگانه ای از جمله اختلالات ایمنولوژیک ، ژنتیک، اندوکرین، سایتولوژیک، عفونت ها و اندومتریوز در سبب شناسی سقط مکرر و خود به خودی نقش دارند (7). غلظت های پایین اسید فولیک نیز با سقط مکرر مرتبط است. اختلال متابولیسم متیونین - هموسیستئین در 20% از موارد دارای NTD و سقط مکرر خود به خودی (Recurrent Spantaneous Abortion) ( RSA ) وجود دارد که می تواند مکانیسم زمینه ای بیماری زایی آنها باشد (1). مطالعات اپیدمیولوژیک موید نقش هموسیستئین در عوارض بارداری (Complications of pregnancy) از جمله قطع جفت (Placental Abruption)، پره اکلامپسی و سقط خود به خود می باشد (8،9). علاوه بر این، هموسیستئین پلاسما یک نشانگر (Marker) حساس وضعیت فولات محسوب می شود (11،10). افزایش غلظت هموسیستئین پلاسما با وضعیت نامناسب کوفاکتورهای ویتامینی (فولات، ویتامین B12 و ویتامین B6) و نقایص ژنتیکی در آنزیمهای دخیل در متابولیسم هموسیستئین مرتبط است. بنابراین هیپرهموسیستئینمی را می توان نتیجه یک تداخل ژن – ماده مغذی دانست (13،12). هیپرهموسیستئینمی (HHcy) و جهش هموزیگوت C677T در ژن 10-5 متیلن تترا هیدروفولات ردوکتاز (MTHFR) با افزایش خطر اتلاف بارداری مکرر مرتبط است. در حال حاضر HHcyبیشتر به عنوان یکی از عواقب وضعیت فولات و نه ویتامین B12 مطرح می باشد (14). پلی مرفیسم های A1298C و C677T ژن آنزیم MTHFR به طور معمول با نقایص متابولیسم هموسیستئین وابسته به فولات مرتبط بوده و به عنوان عوامل خطر اتلاف رویانی مکرر در اوایل بارداری شناخته می شود (15) و برغلظت هموسیستئین تام (tHcy) و پاسخ به درمان پایین آورنده آن اثر می گذارد ( 16). فعالیت این آنزیم در افراد هموزیگوت و هتروزیگوت از نظر جهش C677T، به ترتیب 75% و 35% کاهش می یابد (17). در افراد هموزیگوت از نظر A1298C نیز 40% کاهش فعالیت مشاهده می شود ولی سطح هموسیستئین آنها از افراد طبیعی بالاتر نیست ( 15). به نظر می رسد که کفایت فولات نقش اساسی در بیان فنوتیپی جهش های MTHFR دارا می باشد (18).

چندین مطالعه بر روی رابطه ویتامین های گروه B به خصوص فولات با سقط خود به خودی منتشر شده است ولی داده های موجود در مورد سقط مکرر محدود است (1). در مطالعات پیشین نشان داده شد که مکمل یاری روزانه اسید فولیک در زنان دارای سابقه سقط مکرر بدون علت (unexplained) موجب کاهش قابل ملاحظه هموسیستئین شد (19). همچنین درمان ترکیبی با پردنیزون، آسپرین، فولات و پروژسترون با میزان تولد زنده بالاتر در مقایسه با عدم درمان در زنان دارای سابقه سقط مکرر بدون علت (IRM) مرتبط بود (20). در یکی از مطالعات، تفاوت معنی داری در میزان سقط ها بین گروه مولتی ویتامین (حاویmg 8 اسید فولیک) و گروه عناصر کم یاب مشاهده نشد (21). در مطالعه ی ویتامین شورای تحقیقات پزشکی، میزان تقریبا مشابهی از سقط در گروه اسید فولیک و گروه کنترل مشاهده شد (22). Amin و همکارانش نیز نشان دادند که N-استیل سیستئین می تواند یک درمان موثر بالقوه در بیماران دارای سابقه اتلاف بارداری مکرر بدون علت باشد (23).

استراتژی های درمانی مختلفی در زنان دارای IRM به کار می رود که از این میان می توان به کورتیکواستروئیدها، آسپرین، هپارین و ایمن سازی لکوسیت اشاره نمود (20). شایع ترین درمان به کار رفته برای سقط جنین مکرر تجویز اسید فولیک بوده (24) و در زنان با سابقه ی اتلاف بارداری زود هنگام مفید می باشد (25). به نظر می رسد که مکمل یاری اسید فولیک درمان انتخابی برای کاهش غلظت هموسیستئین تام پلاسما است (31-26). بنابراین تجویز دوزهای درمانی فولات در زنان دارای IRM منطقی به نظر می رسد (20). مکمل یاری با اسید فولیک یا L-MTHF ارزان بوده و هیچ گونه اثرات جانبی عمده ای ایجاد نمی کند (32). در مطالعات گذشته اغلب از مکمل یاری اسیدفولیک استفاده شده است که این فرم فولات نیز می بایست به نوبه خود به متیل تترا هیدو فولات تبدیل شده تا از نظر بیولوژیکی فعال شود (16). نگرانی عمده موجود در ارتباط با دریافت بیش از حد اسید فولیک، احتمال پنهان ماندن علایم هماتولوژیک کمبود ویتامین B12 است که می تواند موجب تاخیر در تشخیص و در نهایت پیشرفت آسیب عصبی شود (33،34). مزیت احتمالی L-MTHF نسبت به اسید فولیک این است که احتمال پنهان ماندن علایم هماتولوژیک کمبود ویتامین B12 حتی با دریافت بالای L-MTHF نیز بعید می باشد. دوز پایین L-5MTHF در کاهش غلظت هموسیستئین در اشخاص سالم، موثرتر از اسید فولیک می باشد. با توجه به اثرات مشابه این دو فرم بر روی عملکرد عروقی و نیز کاهش tHcy به نظر می رسد که هرگونه مزیت بالینی مرتبط با اسید فولیک با استفاده از L-MTHF نیز قابل دستیابی است (35).

ارتباط بالینی بین هیپرهموسیستئینمی و سقط مکرر بدون علت این پرسش را مطرح می کند که مناسب ترین درمان در دسترس برای پیشنهاد به این بیماران کدام است (36). از آنجاییکه تاکنون مطالعه ای در زمینه ی اثرات مکمل یاری L-5MTHF بر روی زنان مبتلا به سقط مکرر صورت نگرفته است، این مطالعه با هدف مقایسه تاثیر اسید فولیک و 5 متیل تترا هیدروفولات بر سطح سرمی فولات، هموسیستئین و میزان سقط در زنان مبتلا به سقط مکرر انجام می شود. امید است نتایج این مطالعه در ارائه درمان موثرتر و کم عارضه برای زنان مبتلا به سقط مکرر موثر باشد.

***7- بررسي متون***

Sutterlin و همکارانش در سال 1997مطالعه ای را با عنوان سطوح سرمی فولات و کوبالامین در زنان دارای سابقه سقط مکرر خود به خودی منتشر کردند. وضعیت فولات و کوبالامین در 29 زن غیرباردار با سابقه ی سقط مکرر خود به خودی (3یا بیش از 3سقط متوالی ) و سبب شناسی ناشناخته با 29 زن سالم بدون سابقه بارداری (Nulligraridae) با سن تولید مثلی (Reproductive age) مشابه مقایسه شد. IRM به صورت 3یا بیش از 3 سقط متوالی در 18 هفته ی اول بارداری با تایید تست بیوشیمیایی بارداری ، سونوگرافی یا آزمایش بافت شناسی تعریف شد. بارداری های نابجا یا خاتمه بارداری انتخابی از مطالعه خارج شدند. زنانیکه پیش از سقط های مکررشان یک نوزاد سالم بدنیا آورده بودند (سقط کننده های ثانویه) و آنهایی که هیچ فرزند در قید حیات نداشتند سقط کننده های اولیه نامیده شدند. نمونه گیری های خونی شش ماه پس از آخرین سقط جمع آوری شدند. هیچ کدام از افراد گروه مطالعه یا کنترل ، اختلال عملکرد اندوکرینی نداشتند یا از بیماری های عفونی، کلیوی، کبدی، صفراوی و گوارشی رنج نمی بردند. بیماران دارای اختلالات عصبی از جمله صرع از مطالعه خارج شدند. افراد گیاه خوار و زنانی که ضدبارداری های خوراکی، مکمل ویتامینی یا هرگونه داروی دیگر در 6 ماه قبل از خون گیری مصرف می کردند که احتمالا با متابولیسم فولات یا کوبالامین تداخل داشت، وارد مطالعه نشدند. کاریوتیپ خون محیطی والدین (Peripheral Blood Karyotype)، ناهنجاری های متعادل (Balanced) کروزومی آنها را به عنوان علت سقط ها رد کرد. ناهنجاری های رحمی شدید با hysteroscopy , hysterosalpingogram یا سطح بالایی از اسکن سونوگرافی رد شدند. اختلالات ایمنولوژیک با تست های آنتی بادی های آنتی کاردیولیپین و آنتی کواگولان لوپوس رد شد. غلظت ویتامین B12 با آزمون های فلورومتریک اتوماتیک اتصال به آنزیم automated fluorometric enzyme – linked assay)) اندازه گیری شد. غلظت های سرمی فولات و کوبالامین بین دو گروه تفاوت معنی داری نداشت. هیچ رابطه ای بین سن و غلظت های ویتامینی یافت نشد. در گروه مطالعه یک رابطه ی معنی دار معکوس بین تعداد سقط های قبلی و غلظت فولات سرم مشاهده شد. بیماران دارای حداقل 4 سقط قبلی به طرز معنی داری مقادیر پایین تر اسید فولیک نسبت به زنان دارای 3 سقط داشتند ولی نسبت به گروه کنترل اینگونه نبود. دلایل زمینه ای این یافته ها نامعلوم است. نتایج این مطالعه نشان داد که غلظت های سرمی فولات و ویتامین B12 در زنان دارای سابقه ی سقط مکرر خود به خودی بدون علت به طرز قابل ملاحظه ای تغییر کرده است و رابطه ای بین کمبود این ویتامین ها و افزایش ریسک اتلاف بارداری در اکثریت بارداری ها مورد تردید به نظر می رسد (1).

Nelen و همکارانش در سال 1998 مطالعه ای را با این عنوان منتشر کردند که پلی مرفیسم MTHFR بر تغییر غلظت های هموسیستئین و فولات ناشی از مکمل یاری اسید فولیک با دوز پایین در زنان با سابقه سقط مکرر بدون علت اثر می گذارد. برای تعیین اثرات مکمل یاری روزانه 5/0 میلی گرم اسید فولیک بر غلظت های هموسیستئین و فولات، 49 زن با سابقه سقط مکرر بدون علت بررسی شدند. زنان مورد مطالعه شامل سقط کننده های اولیه و ثانویه بودند. سقط خود به خود به صورت اتلاف بارداری زود هنگام در خلال 16هفته سن قاعدگی (Menstrual Age) تعریف شد. بنابراین سقط های انتخابی یا عمدی (Elective) و بارداری های نابجا از مطالعه خارج شدند. سقط مکرر (Recurrent Miscarriage) به صورت حداقل 2 سقط خود به خود پس از باردار شدن از یک همسر یکسان تعریف شد. تمام اتلاف های زودرس بارداری به صورت هیستولوژیک یا با یک تست معمول مثبت HCG ادراری (بیش از 50 IU/L ) یا سونوگرافی تایید شدند. در تمام زنان مورد مطالعه، ناهنجاری های کروموزمی، آنومالی های شدید رحمی، اختلال عملکرد تیروئید، عدم تحمل گلوکز و اختلالات ایمونولوژیک با روش های تحقیقاتی معمول بررسی و افراد دارای این شرایط از مطالعه خارج شدند. همچنین زنان مبتلا به کمبود شدید ویتامین B12 (کمتر از 100pmol/L) یا بیماری کبدی یا کلیوی از مطالعه خارج شدند. تمامی زنان وارد شده به مطالعه از سلامت عمومی مناسبی برخوردار بودند. آزمون بارگیری متیونین (Methyonin Load) (شامل غلظت های ویتامینی مورد نظر) قبل و 2 ماه پس از دریافت اسید فولیک انجام شد. در زمان اندازه گیری هیچکدام از زنان باردار یا شیرده نبودند و از قرص های ضد بارداری یا داروهایی که با متابولیسم متیونین-هموسیستئین تداخل دارند، استفاده نمی کردند. هیچکدام از زنان در 6 ماه قبل از اولین اندازه گیری از مکمل ویتامینB استفاده نکرده بودند. تجویز اسید فولیک (5/0 میلی گرم در روز) پس از اولین تست بارگیری متیونین آغاز شد. پایبندی به درمان با کارت های یادداشت (history and diary cards) تایید شد. تعیین PLP (Pyridoxal Phosphate) با تکنیک HPLC (High Perfarmanuy Liquid Chromatography) انجام شد. غلظت ویتامین B12 و فولات بطور همزمان با Dualcount Solid Phase Boil Radio assay اندازه گیری شد. غلظتtHcy با تکنیک HPLC و Fluorinertic detection اندازه گیری شد. جهشC677T با PCR (Polymerase Chain Reaction) یک فراگمنت ژنومیک DNA و سپس آنالیز آنزیم محدود کننده با Hin FI انجام شد. اثرات این مکمل یاری پس از طبقه بندی (Stratification) برای پلی مرفیسم C677T،5-10 MTHFR مورد بررسی قرار گرفت. مکمل یاری اسید فولیک (به مدت 2ماه) موجب کاهش میانه هموسیستئین ناشتا و هموسیستئین دلتا تام پلاسما ( پس از بارگیری منهای ناشتا) به مقدار 27درصد (P<0/001) و 14 درصد (P<0/05) شد. غلظت های میانه ی فولات سرم و گلبول قرمز به ترتیب 275 و 70 درصد افزایش یافت ( P<0/01). اثر پایین آورنده ی هموسیستئین بیش از همه در زنان دارای بالاترین غلظت های تام هموسیستئین درآغاز مطالعه، قابل ملاحظه بود. تمام ژنوتیپ های MTHFR، TT، TC و CC پاسخی متفاوت به مکمل یاری داشتند. پس از 2 ماه زنان هموزیگوت بیشترین کاهش در میانه غلظت های هموسیستئین تام ناشتا (41% P<0/01) و پایین ترین افزایش مطلق در غلظت فولات سرم را نشان دادند (26 نانومول بر لیتر با P<0/05). در نتیجه 2 ماه مکمل یاری روزانه با 5/0 میلی گرم اسید فولیک در زنان با سابقه سقط مکرر بدون علت به طور کلی موجب کاهش قابل ملاحظه غلظت های هموسیستئین تام شد. این اثر بیش از همه در زنان با بالاترین غلظت های هموسیستئین تام در آغاز مطالعه و زنان هموزیگوت از نظر جهش C677T در ژن MTHFR مشهود بود. محدودیت این مطالعه فقدان گروه دارونما بود زیرا در آلمان مکمل یاری اسیدفولیک به تمام زنانی که قصد بارداری دارند توصیه می شود و به همین علت طراحی مطالعه با دارونما از نظر اخلاقی امکانپذیر نیست (19).

Nelen و همکارانش در سال 2000 مطالعه ای را با عنوان سطوح فولات و هموسیستئین به عنوان عوامل خطر اتلاف مکرر در اوایل بارداری منتشر کردند. هدف این مطالعه برآورد خطر نسبی (Relative Risk) (RR) اتلاف مکرر در اوایل بارداری برای غلظت های متفاوت هموسیستئین پلاسما و فولات سرم بود. در این مطالعه مورد- شاهدی غلظت هموسیستئین ناشتا و پس از بارگیریafter load) )، فولات (سرم و گلبول قرمز)، PLP و کوبالامین در 123 زن که حداقل دو اتلاف بارداری خود به خودی زودهنگام به طور متوالی داشتند، اندازه گیری و با غلظت های 104 شاهد سالم مقایسه شد. زنان با اتلاف بارداری مکرر زودهنگام به طور معنی داری غلظت های فولات سرم پائین تری نسبت به شاهدها داشتند. افزایش Hcy ناشتا بیش از 3/18 میکرومول بر لیتر و Hcy پس از بارگیری بیش از 5/61 میکرومول بر لیتر عامل خطر اتلاف بارداری زودهنگام بودند و OR (Odds Ratio) آنها به ترتیب3/6 (CI 1/2-16/7) و 2/7 (CI 0/9-24/3) و 4/3 (CI 1/2-17/3) در سقط کننده های اولیه، و 4/2 (CI 1/3-15/4) و 3/4 (CI 1-12/8) در افراد دارای 3 یا بیش از 3سقط جنین بود. OR ها در جمعیت مطالعه مشابه برای غلظت های فولات سرم کمتر از 4/8 نانومول بر لیتر به ترتیب 1/2 (CI 0/9-4/8) ، 7/2 (CI 1-7/8) و 2/3 (CI 1/3-8/1) بود. رابطه معنی دار دوز پاسخ بین غلظت های فولات سرم و خطر اتلاف بارداری مکرر زودهنگام نشان دهنده اثر حفاظتی غلظت های سرمی بالای فولات بود. افزایش غلظت هموسیستئین و کاهش فولات سرم ریسک فاکتورهای اتلاف بارداری مکرر زودهنگام و خود به خودی بودند. این مطالعه نشان داد که مکمل یاری با فولیک اسید ممکن است در زنان با سابقه اتلاف بارداری زود هنگام مفید باشد (25).

Quere و همکارانش در سال 2001 مطالعه ای را با عنوان مکمل یاری ویتامینی و پیامد بارداری در زنان دارای سابقه اتلاف بارداری زودهنگام و هیپرهموسیستئینمی منتشر کردند. از آنجایی که اسید فولیک و ویتامین B6 می تواند هموسیستئینمی ناشتای تام را به حالت طبیعی در آورند، آنها یک ارزیابی آزمایشی کنترل نشده با مکمل یاری ویتامینی در زنان دارای سابقه اتلاف بارداری زودهنگام مکرر بدون علت و هیپرسیستئینمی انجام دادند. 25 بیمار دارای هیپر هموسیستئینمی با محدوده سنی 27-20 سال که فاقد فرزندان بیولوژیک بودند مورد مطالعه قرار گرفتند. هر کدام از بیماران سابقه 3 تا 5 اتلاف بارداری بین هفته های 8 تا 16 پس از آمنوره در غیاب هر گونه مکمل یاری فولات در طی بارداری داشتند. تست های آزمایشگاهی، استعداد ذاتی یا اکتسابی به ترومبوز را نشان ندادند (سطوح طبیعی آنتی ترومبین، پروتئین C و S، فقدان آنتی بادی های آنتی فسفولیپید و فقدان جهش های فاکتور V یا II لایدن). تمام 25 بیمار از نظر آلل C6177T ژن MTHFR هموزیگوت بودند. ابتدا اثر یک ماه اسیدفولیک با دوز بالا (روزانه 15 میلی گرم) و ویتامین B6(روزانه 750 میلی گرم) را بر غلظت هموسیستئین پلاسما بررسی شد. هموسیستئینمی اکثر بیماران طبیعی شد و بنابراین همین مداخله را در طی بارداری بعدی ادامه دادند. 22 بیمار در طی دوره 3 ماهه پس از طبیعی شدن هموسیستئینمی بارداری دیگری را آغاز کردند. 20 بیمار در 16 هفته پس از آمنوره نیز هنوز باردار بودند. تمامی 20 بارداری بدون هیچ گونه نقص Malformation با موفقیت انجام شد. در چهار مورد تولد زودرس رخ داد که در دو مورد آنها عقب ماندگی رشد جنینی خفیفی رخ داد. اگرچه تکامل پس از زایمان آنها خوب بود. سه ماه بعد از بارداری دریافت ویتامین B6 متوقف شد و دوز روزانه اسید فولیک نیز تا 5 میلی گرم کاهش داده شد. این محققین پیشنهاد کردند که هایپرهموسیستئینمی می تواند به عنوان یک نشانگر در زیرگروهی از زنان محسوب شود که هنگام دریافت مکمل یاری ویتامینی بیشتر مستعد داشتن پیامدهای بارداری مناسب می باشند (36).

Fohr وهمکارانش در سال 2002 مطالعه ای را با این عنوان منتشر کردند که ژنوتیپ 5،10 متیلن تتراهیدروفولات ردوکتاز تعیین کننده اثر پایین آورنده هموسیستئین پلاسمای مکمل یاری با 5 متیل تترا هیدروفولات یا اسیدفولیک در زنان جوان سالم می باشد. هدف این مطالعه ارزیابی اثر احتمالی پایین آورنده tHcy دوز پایین فولیک اسید و یا متیل تتراهیدروفولات با توجه ژنوتیپ MTHFR بود. در این مطالعه تصادفی دو سوکور کنترل شده با دارونما 160 زن شرکت کردند که 400 میکروگرم اسید فولیک و یا مقدار مولی معادل آن متیل تتراهیرو فولات ( 480 میکروگرم مخلوط راسمیک ) و یا دارونما را به صورت روزانه در طی یک دوره 8 هفته ای دریافت کردند. نمونه های خونی در آغاز مطالعه و هفته های 4 و 8 جمع آوری شد. شرکت کنندگان زنان سالم غیر باردار داوطلب 39- 19 ساله بودند . معیارهای ورود شامل، عدم سابقه بیماری اخیر روانی یا جسمی، نتایج طبیعی تست های معمول آزمایشگاهی و وضعیت مناسب ویتامین B12 بود (ویتامین B12 بیشتر و یا مساوی 110 پیکومول بر لیتر). معیارهای خروج اصلی شامل، اعتیاد به الکل یا مواد مخدر، مداخلات درمانی که بر متابولیسم فولات اثر می گذارند و بارداری یا شیردهی بود. از تمامی افراد خواسته شد تا عادات غذایی خود را در طی مدت مطالعه حفظ کنند ولی از دریافت مکمل های ویتامینی یا غذاهای غنی از ویتامین پرهیز کنند. ریزش نمونه احتمالی در این مطالعه 10% در نظر گرفته شد. افرادیکه از داروهای موثر بر متابولیسم فولات استفاده می کردند یا دریافت دارویی دائم خود را تغییر دادند از مطالعه حذف شدند. جمعا 110 نفر در آنالیز وارد شدند. پس از طبقه بندی بر حسب ژنوتیپ، شرکت کنندگان به صورت تصادفی به سه گروه درمانی اختصاص یافتند. در طی دوره درمانی 8 هفته ای از افراد خواسته شد هر روز صبح غیر از روزهای نمونه گیری پیش از صبحانه یک کپسول دریافت کنند ( در روزهای نمونه گیری کپسول پس از نمونه گیری دریافت شد). پای بندی به درمان با شمارش قرص ها ارزیابی شد. در هر مراجعه به مرکز تحقیقات برای جمع آوری نمونه های خونی پای بندی افراد به پروتوکل مطالعه با استفاده از پرسشنامه بررسی شد. هموسیستئین تام و فولات پلاسما در هفته های 4-، صفر، 4 و 8 و فولات گلبول قرمز خون در هفته های صفر و هشت و ویتامین B12 و B6 در آغاز مطالعه( هفته صفر) اندازه گیری شد. متغیرهای خونی با استفاده از تکنیک های آزمایشگاهی استاندارد اتوماتیک اندازه گیری شد. غلظت کراتینین سرم به عنوان نشانگر عملکرد سرم اندازه گیری شد. پای بندی به صورت دریافت ≥ 2 کپسول بیشتر یا کمتر از تعداد تجویز شده در طی مدت 8 هفته تعریف شد. هر دو مکمل فولیک اسید و تترا هیدروفولات ایمن بودند و به خوبی تحمل شدند و هیچ گونه اثرات جانبی ناخوشایند وابسته به درمان مشاهده نشد. مکمل یاری با اسید فولیک موجب کاهش معنی دار غلظت tHcy تا حدود بیشتر و یا مساوی 13% در زنان دارای هر سه ژنوتیپ پس از هفته های 4 و 8 شد. بیشترین کاهش در حدود 20% بود که در زنان دارای ژنوتیپ TT پس از 4 هفته مشاهده شد. مکمل یاری با متیل تترا فولات نیز باعث کاهش tHcy شد که فقط زنان دارای ژنوتیپ CT، کاهش معناداری را پس از 4 هفته نشان دادند. بیشترین کاهش غیر معنی درا ( 15%) در زنان با ژنوتیپ TT پس از 4 هفته مکمل یاری با متیل تتراهیدرو فولات مشاهده شد. نتایج این مطالعه نشان داد که پاسخ به درمان پایین آورنده tHcy با ژنوتیپ MTHFR، تحت تاثیر قرار می گیرد و زنان دارای ژنوتیپ TT، بیش از سایر زنان از مکمل یاری با اسید فولیک یا متیل تترا هیدرو فولات سود می برند (16).

Francoise و همکارانش در سال 2002 مطالعه ای را با عنوان سطوح پایین vit B12 به عنوان یک عامل خطر برای سقط مکرر بسیار زودهنگام منتشر کردند. هدف این مطالعه بررسی ارتباط بین کمبود ویتامین B12 و سقط مکرر زود هنگام (Early Recurrent Abortion) (ERA) یا سقط مکرر بسیار زود هنگام (Very Early Recurrent Abortion) (VERA) حوالی 5 هفته پس از آمنوره بود. زنان دارای ERA قبل از 12 هفته آمنوره و VERA قبل از 6 هفته آمنوره انتخاب شدند. جمعا 110 زن وارد مطالعه شدند. هیچ کدام از این زنان مکمل ویتامین Bدر 6 ماه اخیر دریافت نکرده بودند واز قرص های ضدبارداری خوراکی استفاده نمی کردند. ERA به صورت 2 یا بیش از 2 سقط متوالی با یک همسر یکسان تعریف شد. بارداری ها با تست بارداری بیوشیمیایی (گنادوتروپین کوریونیک انسان سرم ≥ 50IU/L) برای VERA، سونوگرافی یا هیستولوژی تایید شدند. بارداری های نابجا از مطالعه خارج شدند. زنان دارای ناهنجاری های رحمی شدید پس از هیستروسالپینوگرام، هیسترسکوپی یا سونوگرافی سطح بالا از مطالعه خارج شدند. افرادی که وارد مطالعه شدند قند خون و سطح هورمون طبیعی داشتند (هورمون محرک تیروئید، تستسترون و در زنان بالای 38 سال هورمون محرک فولیکول و استرادیول). همگی از نظر بررسی های اتوایمیون (لوپوس آنتی کواگولانت، آنتی کاردیولیپید، آنتی بادی های آنتی DNA و آنتی تیروئید) منفی بودند و از نظر پروتئین C ، S و آنتی ترومبین III طبیعی بودند و به پروتئین C فعال شده و جهش پروترومبینG20210A مقاوم بودند. گروه مورد مطالعه با 96 زن غیر یائسه سالم با یک یا بیش از یک فرزند مقایسه شدند. این زنان نیز سابقه بارداری های طبیعی داشتند و سابقه سقط یا مصرف مکمل ویتامینB در 6 ماه اخیر یا استفاده از قرص های ضدبارداری خوراکی نداشتند همچنین همگی آنها سالم و فاقد بیماری کلیوی، کبدی و عروقی بودند. غلظت فولات و ویتامین B12 سرم با کیت رادیواسی Simul trac-SNB اندازه گیری شد. سطوح هموسیستئین پلاسما نیز با استفاده از آنالیزگر Abbott IMX اندازه گیری شد. 10 زن در مقایسه با یک زن گروه کنترل دارای سطوح پایین ویتامین B12 بودند. 50 درصد این افراد هموسیستئین افزایش یافته داشتند. هیچ تفاوتی بین موردها و شاهدها از نظر فولات مشاهده نشد. ازمیان زنان دارای سطوح پایین ویتامین B12 سرم و ERA ، 5/85% سقط ها VERA بود. مکمل یاری ویتامین B12 منجر به 4 بارداری طبیعی در 5 زن شد که دوباره باردار شده بودند. در یک آنالیز آماری بر روی 5 مطالعه که در آنها ویتامین B12 سرم در ERA بررسی شده بود از جمله این مطالعه، یک ارتباط معنی دار بین کمبود ویتامین B12 و ERA مشاهده شد. نتایج این مطالعه نشان می دهد که کمبود ویتامین B12ممکن است در ERA نقش داشته باشد و ارزیابی ویتامین B12 باید در زنان دارای سابقه ERA صورت گیرد، هرچند ناهنجاری های هماتولوژیک موجود باشد یا خیر (37).

Zetterberg و همکارانش در سال 2002 مطالعه ای را با عنوان افزایش فراوانی ترکیب آلل های جهش یافته C677T و A1298C متیلن تتراهیدروفولات ردوکتاز در رویان های سقط شده به طور خود به خودی منتشر کردند. در این مطالعه شیوع ترکیبی پلی مرفیسم های A1298C و C677T ژن MTHFR در نمونه های DNA رویان های سقط شده به طور خود به خودی (مرگ جنینی بین هفته های 6 تا 20 پس از لقاح) و شاهدهای بزگسال با استفاده از تکنیک ریزتوالی یابی فاز جامد (Solid phase mini sequencing technique) تعیین شد. نمونه ها باPCR amplification و Restriction Fragment Length Polymorphism (RFLP) آنالیز شدند. نسبت شانس قابل ملاحظه 14/2، (CI 1/78_113) در رویان های سقط شده به طور خود به خودی هنگام مقایسه شیوع یک یا بیش از آلل 1298C و 677T در مقایسه ژنوتیپ ترکیبی نوع وحشی (1298AA-677CC) مشاهده شد که نشان دهنده اینست که پلی مرفیسم MTHFR اثر عمده ای بر بقای جنینی دارد. ژنوتیپ های ترکیبی 1298CC-677CT ، 1298AC-677TT یا 1298CC-677TT که حاوی سه یا چهار آلل جهش یافته می باشند در هیچکدام از گروه ها یافته نشد که نشانگر رابطه کامل عدم تعادل بین دو پلی مرفیسم است. یافته های این مطالعه در مورد شیوع بالای ژنوتیپ های جهش یافته ی MTHFR در رویان های سقط شده به طور خود به خودی بر نقش حفاظتی احتمالی مکمل یاری فولیک اسید پیش از لقاح تاکید دارد (15).

Kumar و همکارانش در سال 2003 مطالعه ای را با این عنوان منتشر کردند که سطوح هموسیستئین پلاسما با تداخلات بین وضعیت فولات و جهش ژن متیلن تتراهیدروفولات ردوکتاز در زنان با اتلاف بارداری مکرر بدون علت مرتبط است. از آنجاییکه به نظر می رسد هیپرهموسیستئینمی با هر دو عامل محیطی و ژنتیک تعیین می شود، مطالعه حاضربه منظور یافتن تداخلات بین وضعیت فولات و جهش MTHFR برغلظت هموسیستئین در 24 زن با تجربه 3 یا بیش از 3 اتلاف بارداری مکرر متوالی انجام شد. غلظت میانه هموسیستئین ناشتای پلاسما در گروه مورد مطالعه 23/10 میکرومول بر لیتر و در گروه کنترل 95/8 میکرومول بر لیتر بود(P = 0.096). سطوح افزایش یافته هموسیستئین (> 18 میکرومول بر لیتر) که به عنوان یک ریسک فاکتور اتلاف بارداری مکرر زود هنگام محسوب می شود، در 4زن گروه مطالعه مشاهده شد، در حالیکه در گروه کنترل چنین سطوحی مشاهده نشد. سطوح پایین تر فولات گلبول قرمز(دامنه طبیعی ≥160ng/ml) در 9زن(5/37%) گروه مطالعه در مقایسه با 5 زن(84/20%) گروه کنترل مشاهده شد. میانگین ± انحراف معیار سطوح فولات گلبول قرمز در گروه مطالعه07/37± 37/154 بود در حالیکه در گروه کنترل برابر با 97/28± 0/159 بود. در مطالعه حاضر 6زن گروه مطالعه و 2زن گروه کنترل ناقل جهش C677T MTHFR بودند. هیچ کدام از زنان برای آلل های جهش یافته (TT) هموزیگوت نبودند. بالاترین مقادیر غلظت هموسیستئین در زنان با سابقه اتلاف بارداری مکرر دارای کمبود فولات به همراه ژنوتیپ CT مشاهده شد. نتایج این مطالعه حاکی از آنست که تعیین هیپرهموسیستئینمی در زنان با اتلاف بارداری مکرر ممکن است به نرمال سازی درمانی کمک کرده و شاید تولد طبیعی را میسر سازد (38).

Venn و همکاران در سال 2003 مطالعه ای را با عنوان مقایسه اثرات مکمل یاری با دوز پایین L_5MTHF یا اسید فولیک بر هموسیستئین منتشر کردند. این مطالعه به صورت یک مداخله تصادفی کنترل شده با دارونما به مدت 24 هفته طراحی شده بود. هدف این مطالعه مقایسه اثر بخشی µg 100 فولیک اسید در روز یا معادل مولی ((equimolar مشابه L-5MTHF در کاهش tHcy در داوطبان سالم بود. افراد مورد مطالعه هیچگونه مکمل حاوی اسید فولیک مصرف نمی کردند و دریافت بالایی از غذاهای غنی شده با فولات نداشتند (به عبارت دیگر کمتر از 3 سروینگ در هفته). افراد دارای بیماری های مزمن، و آنهایی که باردار بوده یا قصد بارداری داشتند وارد مطالعه نشدند. داوطلبان سالم (n=167) به طور تصادفی برای دریافت روزانه مکمل حاوی اسید فولیک (gµ100)، L_5MTHF ( µg 113) یا دارونما تقسیم تصادفی شدند. نمونه های خونی در آغاز مطالعه و در هفته های 8، 16 و 24 جمع آوری و از نظر غلظت tHcy، فولات پلاسما و فولات گلبول قرمز (Red Blood Cell Folat) (RCF) مورد بررسی قرار گرفتند. یک پرسشنامه دموگرافیک و شیوه زندگی و نیز یک پرسشنامه خوراک 63 ایتمی که به منظور ارزیابی دریافت فولات معمول افراد در ماه گذشته طراحی شده بود، تکمیل شد. میزان پایبندی افراد با شمارش مکمل های بازگشت داده شده و بررسی فرم های یادداشت تکمیل شده ارزیابی شد. غلظت ویتامین B12 تنها درآغاز مطالعه اندازه گیری شد ولی فولات پلاسما، فولات گلبول قرمز (RCF) و tHcy در آغاز مطالعه و هفته های 8، 16 و24 اندازه گیری شد. پس از تطابق، برای مقادیر آغاز مطالعه، در هفته 24مطالعه میانگینtHcy در گروه L_5MTHF و اسید فولیک، به ترتیب 6/14% و 3/9% پایین تر، میانگین فولات پلاسما 34% و 52% بالاتر و میانگین RCF آنها 23% و 31% بالاتر از گروه دارونما بود. L-5MTHF نسبت به اسید فولیک در پایین آوردن tHcy موثرتر بود (P< 0/05). در هفته بیست وچهارم افزایش غلظت فولات پلاسما و RCF بین دو گروه مکمل یاری شده تفاوت معنی داری نشان نداد. نتایج این مطالعه نشان داد که دوز پایین L-5THF در کاهش غلظت هموسیستئین در اشخاص سالم موثرتر از اسید فولیک می باشد (35).

Tempfer و همکارانش در سال 2006 مطالعه ای را با عنوان یک درمان ترکیبی با پردنیزون، آسپرین، فولات و پروژسترون در زنان با سابقه سقط مکرر ایدیوپاتیک (بدون علت) منتشر کردند. این کارآزمایی بالینی از طراحی Matched Paired برخوردار بود. هدف این مطالعه مقایسه درمان ترکیبی با پردنیزون، آسپرین، فولات و پروژسترون با عدم درمان در زنان دارای سابقه سقط مکرر ایدیوپاتیک (IRM) بود. افراد مورد مطالعه شامل زنان دارای سابقه IRM بود که به صورت 3 یا بیش از 3 سقط متوالی قبل از هفته بیستم بارداری بدون پاتولوژی های آناتومیک، سیتولوژیک، هورمونی و عفونی یا سندرم آنتی فسفولیپید تعریف شدند. 80 نفر از 210 زن واجد شرایط موافقت خود را برای شرکت در مطالعه اعلام کردند و با پردنیزون (mg/day 20) و پروژسترون (mg/day 20) برای 12هفته اول بارداری، آسپرین (mg/day 100) برای 38 هفته بارداری و فولات (mg 5) هر دو روز (every second day) در طی بارداری شان تحت درمان گرفتند. 52 نفر از 80 زن باردار شدند و با 52 زن دارای IRM (همسان شده از نظر سن و تعداد سقط ها) که بدون درمان در طی دوره یکسان مشاهده شده باردار شدند، مقایسه شدند. معیارهای پیامد اصلی شامل میزان تولد زنده، عوارض بارداری ازجمله پره اکلامسی، تولد نارس و محدودیت رشد داخل رحمی و اثرات جانبی مرتبط با درمان بود. میزان تولد زنده کلی گروه های درمان وکنترل 77% (52/40) و 35% (52/18) بود (P = 0/04). میزان های سقط سه ماهه ی اول و دوم در میان گروه های درمان وکنترل 19% (52/10) و صفر% (52/صفر) و 63% (52/33) و 2% (52/1) بود (به ترتیب P =1, P = 0/09). میانه سن بارداری در هنگام تولد و میانه وزن تولد بین گروه های درمان تفاوتی نداشت. دو و سه مورد تولد نارس در میان گروه های درمان و کنترل مشاهده شد ( P = 0/3) و هیچ مورد محدودیت رشد درون رحمی و بیماری CUHSING مشاهده نشد. از 80 زنی که درمان را شروع کردند یک زن بارداری نابجا داشت و یک زن بارداری خود را به علت اختلال کروموزمی جنین خاتمه داد ( تری زومی 18)، 3 زن به علت تهوع، افسردگی و تاکی کاردی درمان را متوقف کردند. این مطالعه نشان داد که یک درمان ترکیبی با پردنیزون، آسپرین، فولات و پرژسترون با میزان تولد زنده بالاتر در مقایسه با عدم درمان در زنان دارای سابقه IRM مرتبط است. نتایج این مطالعه باید با احتیاط تفسیر شود. اولا تعداد زنان وارد شده به مطالعه پایین بود و امکان حذف تفاوت های کوچک در اثرات جانبی ناخواسته را میسر نمی سازد. بنابراین اگرچه یک اثر مثبت از این درمان ترکیبی در این نمونه خاص بیماران مشاهده شد، امنیت این درمان با توجه به این مطالعه غیرقابل بررسی است. محدودیت دیگر این مطالعه، طراحی matched paired بود. تورش انتخاب احتمالی در جداسازی بیماران گروه درمان و کنترل می تواند در این طراحی مطالعه آشکار (open) ایجاد شده باشد. همچنین این مطالعه به علت عدم Karyotype کردن بافت بارداری سقط شده قادر به تمایز بین سقط های euploid و aneuploid نیست. اگر زنان دارای سقط aneuploid از مطالعه خارج می شدند، کارآیی درمان بالاتر بود (20).

Nadir و همکارانش در سال 2007 مطالعه ای را با عنوان ارتباط هموسیستئین، ویتامین B12، اسیدفولیک و MTHFR C677T در بیماران دارای سابقه ترومبوز یا اتلاف جنینی مکرر منتشر کردند. هدف این مطالعه ارزیابی رابطه بین این چهار پارامتر در بیماران دارای سابقه ترومبوز یا اتلاف جنینی مکرر بود. جمعیت مورد مطالعه شامل 326 بیمار بود که به بخش ترومبوز و هموستاز ارجاع می شدند. 120 نفر از این بیماران حداقل دارای یک نتیجه آزمایش پاتولوژیک برای این چهار پارامتر بودند. تست های خونی حداقل سه ماه پس از اتلاف جنینی یا حادثه ترومبوتیک انجام شدند. بیماران دارای نتایج غیر طبیعی آزمونهای عملکرد کلیوی یا بدخیمی های فعال از مطالعه خارج شدند. 51 زن به علت اتلاف جنین مکرر مورد ارزیابی قرار گرفتند. اتلاف جنینی مکرر به صورت سه و یا بیش از سه اتلاف جنینی در سه ماهه اول، دو و یا بیش از دو اتلاف جنینی در سه ماهه دوم و یک و یا بیش از یک مرگ جنینی داخل رحمی در سه ماهه سوم تعریف شد. سطوح اسید فولیک و ویتامین B12 سرم به ترتیب با استفاده از سیستم Abbott AxSYM و بر اساس تکنولوژی اتصال یون و تکنولوژی Microparticles Enzyme Immunoassay ارزیابی شد. سطح هموسیستئین ناشتای پلاسما نیز فورا با Fluorescence Polarization Immunoassay و بر روی یک آنالیزگر Abbott IMX اندازه گیری شد. آنالیز جایگزینی C677T ژن MTHFR با تقویت فراگمنت های 198 جفت بازی DNA و سپس هضم آنها توسط Hinf1 مطابق با روش فراست و همکارانش انجام شد. رابطه بین هموسیستئین و ویتامین B12 و نیز رابطه هموسیستئین و فولیک اسید ضعیف بود (r = -0.20, r = -0.23 به ترتیب). تفاوت معنی داری بین میانگین سطح هموسیستئین و ژنوتیپ های CC،CT ، TT و ژن MTHFR مشاهده نشد (p= 0.24). در نتیجه در جمعیت مورد مطالعه رابطه بین هموسیستئین، ویتامین B12، اسیدفولیک و ژنوتیپ MTHFR C677T ضعیف بود. نتایج این مطالعه، این امر را مورد تردید قرار می دهد که ارزیابی معمول کنونی HHC به عنوان جزئی از بررسی کامل ترومبوفیلی به واقع نمایانگر افزایش خطر ترومبوز باشد. محدودیت های این مطالعه تعداد محدود بیماران ارزیابی شده و نیز فقدان گروه کنترل بود (32).

Amin و همکارانش در سال 2008 مطالعه ای را با عنوان N استیل سیستئین برای درمان اتلاف بارداری مکرر بدون علت منتشر کردند. از آنجایی که بارداری می تواند با حالتی از استر س اکسیداتیو مرتبط باشد که قادر است آبشاری از تغییرات را آغاز، تسریع و منجر به شکست بارداری شود، آنها چنین فرض کردند که این فرآیند استرس اکسیداتیو ممکن است با اثر آنتی اکسیدانی n- استیل سیستئین سرکوب شود. هدف این مطالعه ارزیابی اثر درمان با N- استیل سیستئین در بیماران دارای سابقه اتلاف بارداری مکرر بدون علت بود. این مطالعه آینده نگر کنترل شده در مصر انجام شد. یک گروه 80 نفره از بیماران دارای سابقه اتلاف بارداری مکرر بدون علت با 6/0 گرم N- استیل سیستئین به همراه 500 میکروگرم فولیک اسید مکمل یاری شدند و گروه کنترل که از نظر سنی با گروه درمان تطبیق داده شده بودند، شامل 86 بیمار بود که تنها با 500 میکروگرم فولیک اسید در روز درمان می شدند. N- استیل سیستئین به همراه اسید فولیک نسبت به اسید فولیک به تنهایی موجب افزایش معنی دار میزان تداوم یک بارداری زنده تا 20 هفتگی و بیشتر شد. N- استیل سیستئین به همراه اسید فولیک موجب افزایش معنی دار میزان فرزندانی که به خانه ارجاع داده می شدند نسبت به اسید فولیک به تنهایی شد. نتایج این مطالعه نشان داد که N- استیل سیستئین دارویی است که به خوبی تحمل می شود و می تواند یک درمان موثر بالقوه در بیماران دارای سابقه اتلاف بارداری مکرر بدون علت باشد (23).

به طور کلی بازنگری مطالعات پیشین نشان داد که اولا تا کنون مطالعه ای در زمینه اثرات مکمل 5 متیل تتراهیدروفولات بر روی زنان مبتلا به سقط مکرر صورت نگرفته است، اما در مطالعات اندکی که در زمینه اثرات مکمل اسید فولیک در این افراد صورت گرفته است، مکمل اسید فولیک موجب افزایش فولات پلاسما و میزان موفقیت در بارداری و کاهش میزان هموسیستئین پلاسما، سقط خود به خود و برخی دیگر از عوارض بارداری شده است. ثانیا مکمل 5 متیل تتراهیدروفولات در افراد سالم موجب افزایش فولات پلاسما و کاهش میزان هموسیستئین پلاسما شده بود که این اثرات نسبت به مکمل اسید فولیک مشهودتر بود. ثالثا تا کنون هیچ مطالعه ای در زمینه مقایسه تاثیر اسید فولیک و 5- متیل تترا هیدروفولات بر سطح سرمی فولات، هموسیستئین و میزان سقط در زنان مبتلا به سقط مکرر انجام نشده است.

***8- اهداف و فرضيه‌ها***

**هدف کلی :**

مقایسه تاثیر اسید فولیک و 5- متیل تترا هیدروفولات بر سطح سرمی فولات، هموسیستئین و میزان سقط در زنان مبتلا به سقط مکرر

**اهداف اختصاصی :**

- تعیین متغیرهای سن، وزن، قد، نمایه توده بدن، و تعداد سقط مکرر قبل از آغاز مطالعه، وضعیت سقط زنان مورد مطالعه در دو گروه در زمان شروع مطالعه
- تعیین ژنوتیپ C677T ژن MTHFR، و ژنوتیپ A1298C ژن MTHFR در دو گروه در زمان شروع مطالعه
- تعیین متغیرهای تعداد حاملگی بیشتر از 20 هفته پس از آغاز مطالعه، تعداد سقط کمتر از 20 هفته پس از آغاز مطالعه، تعداد هفته بارداری قبل از زایمان یا سقط در زنان مبتلا به سقط مکرر در دو گروه مورد مطالعه
- تعیین متغیرهای هموسیستئین پلاسما، و فولات پلاسما در زمان شروع و هفته هشتم مطالعه و نیز هفته ششم، دوازدهم و بیستم حاملگی در دو گروه مورد مطالعه.
- مقایسه ميانگين تعداد حاملگی بیشتر از 20 هفته پس از آغاز مطالعه، تعداد سقط کمتر از 20 هفته پس از آغاز مطالعه، تعداد هفته بارداری قبل از زایمان یا سقط در پایان مطالعه بین دو گروه مورد مطالعه.
- مقایسه میانگین متغیرهای هموسیستئین پلاسما و فولات پلاسما در زمان شروع و هفته هشتم مطالعه و نيز هفته ششم، دوازدهم و بیستم حاملگی بین دو گروه مورد مطالعه و در هر گروه بین زمان های مطالعه.
- مقایسه میانگین تغییرات هموسیستئین پلاسما و فولات پلاسما در طول مدت مطالعه بین دو گروه مورد بررسی.

**هدف کاربردی**

ارائه درمان موثرتر و کم عارضه برای زنان مبتلا به سقط مکرر

**فرضیات مطالعه**

- میانگین سن، وزن، قد، نمایه توده بدن، و تعداد سقط مکرر قبل از آغاز مطالعه، وضعیت سقط زنان مورد مطالعه در دو گروه در زمان شروع مطالعه به چه میزان است؟
- شیوع ژنوتیپ C677T ژن MTHFR، و ژنوتیپ A1298C ژن MTHFR در دو گروه در زمان شروع مطالعه به چه میزان است؟
- میانگین تعداد حاملگی بیشتر از 20 هفته پس از آغاز مطالعه، تعداد سقط کمتر از 20 هفته پس از آغاز مطالعه، تعداد هفته بارداری قبل از زایمان یا سقط در زنان مبتلا به سقط مکرر در دو گروه مورد مطالعه متفاوت می باشد
- میانگین هموسیستئین پلاسما، و فولات پلاسما در زمان شروع و هفته هشتم مطالعه و نیز هفته ششم، دوازدهم و بیستم حاملگی در دو گروه مورد مطالعه متفاوت می باشد
- میانگین تغییرات هموسیستئین پلاسما و فولات پلاسما در طول مدت مطالعه بین دو گروه مورد بررسی متفاوت می باشد

***9- متغيرهاي تحقيق***

*متغيرهاي اصلي و مهم را در جدول زير وارد كنيد.*

| مشخصات متغیر  نام متغیر | مستقل | وابسته | مخدوش کننده | **متغیرهای مطالعه:**  **جدول متغیرها**  کمی | | کیفی | | تعریف عملی متغیر | واحد یا مقیاس اندازگیری | ابزار یا روش اندازگیری |
| --- | --- | --- | --- | --- | --- | --- | --- | --- | --- | --- |
|  |  |  |  | پیوسته | گسسته | اسمی | رتبه ای |  |  |  |
| نوع مکمل دریافتی گروه مورد مطالعه | ***** |  |  |  |  | ***** |  | 1- اسیدفولیک (دریافت خوراکی روزانه 5 میلی گرم)  2-5-متیل تتراهیدروفولات (دریافت خوراکی روزانه 5 میلی گرم) | 1-بله  2-خیر | فرم جمع آوری داده ها |
| سن |  |  | ***** |  | ***** |  |  | تعداد سالهاي سپري شده بعد از تولد هنگام پر کردن پرسشنامه | سال | پرونده پزشکی و پرسشنامه اطلاعات عمومی |
| هموسیستئین پلاسما |  | ***** |  | ***** |  |  |  | غلظت هموسیستئین پلاسما | میکرومول بر لیتر | ELISA |
| فولات پلاسما |  | ***** |  | ***** |  |  |  | غلظت فولات پلاسما | نانومول بر لیتر | RIA |
| ژنوتیپ C677T |  |  | ***** |  |  | ***** |  | ژنوتیپ جهش C677T ژن  MTHFR | 1-هموزیگوت ((TT  2-هتروزیگوت ((CT  -3طبیعی ((CC | PCR,RFLP |
| ژنوتیپ A1298C |  |  | ***** |  |  | ***** |  | ژنوتیپ جهش A1298C ژن  MTHFR | 1-هموزیگوت ((CC  2-هتروزیگوت ((AC  -3طبیعی ((AA | PCR,RFLP |
| تعداد سقط مکرر قبل از آغاز مطالعه |  |  | * |  | * |  |  | تعداد سقط مکرر هر زن قبل از آغاز مطالعه | بار | پرونده پزشکی و پرسشنامه اطلاعات عمومی |
| وضعیت سقط زنان مورد مطالعه |  |  | * |  |  | * |  | طبقه بندی زنان به صورت سقط کنندگان اولیه یا ثانویه | 1- اولیه: زنانیکه پیش از سقط های مکررشان هیچ نوزاد سالمی بدنیا نیاورده باشند.  2- ثانویه: زنانیکه پیش از سقط های مکررشان حداقل یک نوزاد سالم بدنیا آورده باشند. | پرونده پزشکی و پرسشنامه اطلاعات عمومی |
| تعداد حاملگی بیشتر از 20 هفته پس از آغاز مطالعه |  | ***** |  |  | ***** |  |  | تعداد حاملگی بیشتر از 20 هفته پس از آغاز مطالعه | عدد | علایم بالینی، تست های بیوشیمیایی، سونوگرافی |
| تعداد سقط قبل از 20 هفته پس از آغاز مطالعه |  | ***** |  |  | ***** |  |  | تعداد سقط قبل از 20 هفته پس از آغاز مطالعه | عدد | علایم بالینی، تست های بیوشیمیایی، سونوگرافی |
| تعداد هفته بارداری قبل از زایمان یا سقط |  | ***** |  | ***** |  |  |  | تعداد هفته بارداری قبل از زایمان یا سقط | عدد | علایم بالینی، تست های بیوشیمیایی، سونوگرافی |
| فولات دریافتی از رژیم غذایی |  |  | * | * |  |  |  | میزان فولات دریافتی از رژیم غذایی | میکرو گرم در روز | پرسشنامه یادآمد خوراک |
| استعمال دخانیات |  |  | * | ***** |  |  |  | مصرف متوسط پاكت سيگار در يك روز (در طول عمر) ضرب در سالهاي مصرف | پاكت-سال (pack-year) | پرسشنامه اطلاعات عمومی |
| وزن |  |  | ***** | ***** |  |  |  | وزن فرد با لباس سبک و بدون كفش با دقت 100 گرم | کیلوگرم | ترازوی دیجیتالی |
| قد |  |  | ***** | ***** |  |  |  | قد فرد در حالت ايستاده و بدون كفش با دقت 1/0 سانتيمتر | سانتی متر | متر نواری نصب شده بر روی دیوار |
| نمايه توده بدن(BMI) |  |  | ***** | ***** |  |  |  | نسبت وزن بر حسب کيلوگرم به توان دوم قد بر حسب متر | کیلوگرم بر متر مربع | محاسبه |

*10- جامعه و نمونه مورد بررسي*

**نوع مطالعه:**

مطالعه حاضر یک کارآزمایی بالینی تصادفی دو سو کور (Double Blind Randomized Clinical Trial) می باشد.

**جامعه هدف:**

زنان مبتلا به سقط مکرر

**نمونه مورد مطالعه:**

در این تحقیق نمونه های مورد مطالعه از میان زنان مبتلا به سقط مکرر مراجعه کننده به مركز فوق‌تخصصی درمان ناباروری و سقط مكرر ابن‌سينا مطابق با معیار های زیر انتخاب می شوند:

**الف- معیارهای ورود به مطالعه**

- تمایل به همکاری و تکمیل فرم رضایت نامه آگاهانه.

- حداقل 6 ماه از آخرین سقط آنها گذشته باشد.

- حداقل سابقه سه یا بیش از سه سقط جنین متوالی خود به خودی از یک همسر داشته باشند.

- عدم دریافت مکمل های حاوی اسيد فوليك در 6 ماه اخیر یا دریافت بالا (بیش از دو سروینگ در هفته) از غذاهای غنی شده با فولات.

- سقط های افراد فاقد پاتولوژی آناتومیک، سیتولوژیک، هورمونی، عفونی یا سندرم آنتی فسفولیپید باشد.

- عدم داشتن رژیم غذایی خاص از جمله گیاهخواری.

**ب- معیارهای خروج از مطالعه**

- سقط های عمدی یا انتخابی (elective)، و حاملگی های نابجا (ectopic).

- وجود بدخیمی های فعال، ناهنجاری های کروموزمی، آنومالی های شدید رحمی، اختلال عملکرد تیروئید، اختلال عملکرد کلیوی یا کبدی، عدم تحمل گلوکز، صرع، اختلالات اندوکرین، ایمونولوژیک، صفراوی و گوارشی، مصرف الکل یا مواد مخدر.

- مصرف قرص های ضدبارداری خوراکی یا داروهایی که بر متابولیسم متیونین- هموسیستئین، فولات اثر می گذارند.

- تغییر در پس زمینه دارویی.

**حجم نمونه و روش نمونه گیری :**

(0.05=α) و توان 80 درصد (0.20=β) از نظر آماری معنی دار گردد. در این مطالعه تعداد نمونه تعداد نمونه لازم جهت انجام این مطالعه بر مبنای متغیر وابسته غلظت هموسیستئین پلاسما محاسبه شد. تعیین حجم نمونه برای این مطالعه بر این اساس بود که چه تعداد نمونه باید انتخاب شوند تا در صورتیکه تفاوت میانگین غلظت هموسیستئین پلاسما بین گروه دریافت کننده مکمل 5 متیل تترا هیدرو فولات از گروه دریافت کننده مکمل اسید فولیک حداقل 1.5 میکرومول بر لیتر باشد، این تفاوت با احتمال 95 درصد برای هر یک از گروه ها 27 نفر برآورد گردید که با توجه به 10 درصد ریزش احتمالی نمونه ها، در هر یک از گروهها 30 نفر در نظر گرفته شد.

حجم نمونه با استناد به انحراف معیارهای بدست آمده از مطالعات قبلی (16) و با استفاده از فرمول زیر محاسبه گردید (40):


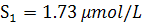


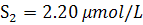


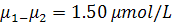


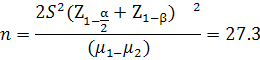


در این مطالعه نمونه ها از مركز فوق‌تخصصي درمان ناباروري و سقط مكرر ابن‌سينا با روش نمونه گیری آسان (Convenience Sampling) انتخاب می گردند (41).

**11- روش و نحوه اجراي تحقيق**

در این مطالعه ابتدا برای زنان مبتلا به سقط مکرر مراجعه کننده به مرکز فوق‌ تخصصی درمان ناباروری و سقط مكرر ابن‌سينا که دارای معیارهای ورود به مطالعه می باشند، موضوع، اهداف و روش اجرای مطالعه توضیح داده می شود. آنگاه در صورت تمایل بیماران به شرکت در این مطالعه از آنها رضایتنامه آگاهانه کتبی از خودشان و همسرانشان اخذ می گردد (پیوست 1). سپس زنان مورد مطالعه به طور تصادفی و با کدهای کامپیوتری يا جدول اعداد تصادفی به 2 گروه دریافت کننده مکمل اسید فولیک و يا مکمل 5 متیل تتراهیدروفولات تقسیم می شوند. به زنان گروه دریافت کننده مکمل اسید فولیک در زمان شروع مطالعه چند جعبه حاوی قرص های مکمل اسید فولیک و به زنان گروه دریافت کننده مکمل 5 متیل تتراهیدروفولات، چند جعبه حاوی قرص های مکمل 5 متیل تتراهیدروفولات داده می شود. از تمامی بیماران خواسته می شود تا در ویزیت های بعدی، جعبه قرص های خود را برای اطمینان از مصرف کامل با خود همراه داشته باشند. جهت دوسوکور اجرا کردن این تحقیق، در زمان شروع مطالعه مجموعه جعبه های حاوی قرص های مربوطه توسط فردی غیر از پژوهشگر بصورت A و B کدگذاری می شوند تا عدم اطلاع محقق از نوع قرص های دریافتی توسط هر گروه رعایت شود. زنان در گروه دریافت کننده مکمل اسید فولیک روزانه 5 میلی گرم (11325 نانو مول) (یک قرص) مکمل اسید فولیک و زنان گروه دریافت کننده مکمل 5 متیل تتراهیدروفولات روزانه 5 میلی گرم (11325 نانو مول) (یک قرص) مکمل 5 متیل تتراهیدروفولات در طول این مطالعه 7 ماهه دریافت خواهند کرد. در شروع و هفته هشتم مطالعه و نیز هفته ششم، دوازدهم و بیستم حاملگی که به طور روتین توسط مرکز درمان ابن سینا خونگیری می شود، غلظت هموسیستئین و فولات پلاسما نیز به ترتیب با روش RIA(SimulTRAC-SNB;ICN)و ELISA(Biosystemt) اندازه گیری می شود. در پایان هفته هشتم مطالعه کلیه زنان دوباره ویزیت شده و به زنان هر گروه چند جعبه حاوی قرص های مکمل داده می شود و از آنها خواسته می شود تا اقدام به حاملگی نمایند (افرادی که وقوع بارداری در آنها بیش از 4 ماه به طول انجامد از مطالعه خارج می شوند)، سپس در هفته های ششم، دوازدهم و بيستم حاملگی یا زمان سقط، تمامی زنان دوباره ویزیت شده و پس از دریافت جعبه های حاوی مکمل، نمونه های خونی و اطلاعات مورد نیاز جمع آوری می گردد. پیگیری بیماران به منظور کنترل آنها از نظر مصرف قرص های اسید فولیک و 5 متیل تتراهیدروفولات، پاسخ به سوالات مربوط به مطالعه آنها و جلوگیری از ریزش نمونه ها از طریق مراجعه بیماران به مرکز فوق ‌تخصصی درمان ناباروری و سقط مكرر ابن‌سينا و نیز هر هفته به صورت تلفنی صورت می گیرد. در این مطالعه ارزشیابی میزان پایبندی بیماران و رعایت پروتوکل مطالعه از نظر مصرف قرص های اسید فولیک و 5 متیل تتراهیدروفولات با تعیین تعداد قرص های اسید فولیک و 5- متیل تتراهیدروفولات باقیمانده در پایان مطالعه و فرم های يادداشت تكميل شده انجام می شود. همچنین در این تحقیق وزن هر بیمار با لباس سبک و بدون کفش و با استفاده از ترازوی دیجیتال seca)) با دقت 100 گرم و قد فرد در حالت ایستاده و بدون کفش توسط متر نصب شده بر روی دیوار با دقت 1/0 سانتی متر در شروع و هفته هشتم مطالعه و نیز هفته ششم، دوازدهم و بیستم حاملگی اندازه گیری می شود. BMI از تقسیم وزن (کیلوگرم) بر مجذور قد (متر مربع) محاسبه می شود. اطلاعات مورد نیاز در مورد سن، استعمال دخانیات، تعداد سقط مکرر قبل از آغاز مطالعه، وضعیت سقط زنان مورد مطالعه در زمان شروع مطالعه با تکمیل پرسشنامه اطلاعات عمومی (پیوست 2) با مصاحبه حضوری و بررسی پرونده های پزشکی بدست می آید. همچنین فولات دریافتی از رژیم غذایی با استفاده از پرسشنامه یادآمد خوراک در مورد 3 روز هفته (2 روز میان هفته و 1 روز آخر هفته) (پیوست 3) با مصاحبه حضوری در آغاز مطالعه و هفته هشتم و نیز هفته ششم، دوازدهم و بیستم حاملگی ارزیابی می شود. آنگاه اطلاعات بدست آمده از طریق این پرسشنامه ها با استفاده از نرم افزار تغذیه ای Nutritionist 4 مورد تجزیه و تحلیل قرار می گیرد. تعیین ژنوتیپ C677T ژن MTHFR و ژنوتیپ A1298C ژن MTHFR نیز در شروع مطالعه با روش PCR ,RFLP انجام می شود. همچنین در پایان این مطالعه ميانگين تعداد حاملگی بیشتر از 20 هفته پس از آغاز مطالعه، تعداد سقط کمتر از 20 هفته پس از آغاز مطالعه، تعداد هفته بارداری قبل از زایمان یا سقط بین دو گروه مورد مطالعه مقایسه می شود. تایید حاملگی ها، سقط ها و تعداد هفته های بارداری با بررسی علایم بالینی، تست های بیوشیمیایی و سونوگرافی انجام خواهد شد. در این مطالعه میزان تحمل و اثرات جانبی احتمالی افراد و نیز پایبندی به پروتوکل مطالعه در رابطه با دریافت مکمل های اسید فولیک و 5 متیل تتراهیدروفولات در هر ویزیت بررسی می شود.

روش تجزیه و تحلیل آماری داده ها :

در این مطالعه، تجزیه و تحلیل آماری داده ها توسط نرم افزار SPSS نسخه 16 صورت می گیرد. جهت مقایسه متغیر های کیفی مخدوش کننده بین دو گروه از آزمون Chi Square استفاده می شود. جهت مقایسه میانگین متغیرهای کمی مخدوش کننده آنتروپومتریک و رژیمی و سایر متغیرهای کمی که در طول مطالعه پنج بار اندازه گیری می شوند، در هر گروه از آزمون آنالیز واریانس برای داده های تکراری استفاده می شود.

**12- ملاحظات اخلاقی پژوهش**

از آنجایی که مکمل یاری با فولیک اسید یا 5 متیل تتراهیدروفولات هیچ گونه اثرات جانبی عمده ای ایجاد نمی کند (32) و شایع ترین درمان به کار رفته برای سقط مکرر جنین تجویز اسید فولیک است (24) و ممکن است در زنان با سابقه ی اتلاف بارداری زود هنگام مفید باشد (25)، لذا انجام این مطالعه از نظر اخلاقی فاقد اشکال می باشد. در این تحقیق جهت رعایت اصول اخلاقی از بیماران داوطلب وارد شده به مطالعه و همسرانشان برگه رضایتنامه آگاهانه کتبی اخذ می گردد (پیوست 1).

**13- جدول‌های تو خالی اصلی**

جدول 1- میانگین و انحراف معیار غلظت همو سیستئین پلاسما و تغییرات آن در زنان مورد مطالعه

| فراسنج  گروه | غلظت هموسیستئین پلاسما ( میکرومول بر لیتر ) | | | | | تغییرات در کل دوره مطالعه | |
| --- | --- | --- | --- | --- | --- | --- | --- |
|  | شروع مطالعه | هفته هشتم مطالعه | هفته ششم حاملگی | هفته دوازدهم حاملگی | هفته بیستم حاملگی | میزان تغییرات | درصد |
| اسید فولیک  (n= ) |  |  |  |  |  |  |  |
| متیل تترا هیدرو فولات  (n= ) |  |  |  |  |  |  |  |

جداول توخالی برای سایر متغیر های اندازه گیری شده در این مطالعه، مشابه با جدول توخالی فوق کشیده می شود.

**14- مشكلات و محدوديتها**

*با توجه به اینکه هر بیمار حداقل 7 ماه باید پیگیری شود، احتمال ریزش نمونه ها وجود دارد که به این منظور تعداد نمونه ها در ابتدا دو برابر در نظر گرفته می شود.*

**15- جدول زمان بندي برنامه تحقيق**

*شامل مهمترين مراحل طرح و زمان ارائه گزارشهای پيشرفت (حداقل 3 گزارش پيشرفت)*

|  |  | | فعاليت های اجرايي | | زمان اجرا (ماه) | | | | | | | | | | | | | | | | | |
| --- | --- | --- | --- | --- | --- | --- | --- | --- | --- | --- | --- | --- | --- | --- | --- | --- | --- | --- | --- | --- | --- | --- |
| 1 | | هماهنگي با رياست و سرپرست مركز فوق ‌تخصصی درمان ناباروري و سقط مكرر ابن‌سينا و تهیه داروها | | ٭ | | ٭ |  |  |  |  |  |  |  |  |  |  |  |  |  |  |  |  |
| 2 | | انتخاب نمونه مورد نیاز از ميان زنان مبتلا به سقط مکرر مراجعه کننده به مركز فوق‌ تخصصی درمان ناباروري و سقط مكرر ابن‌سينا، انجام مداخله، جمع آوری نمونه های خونی و انجام اندازه گیری های آزمایشگاهی، اندازه گیری شاخص های تن سنجی و تکميل پرسشنامه های اطلاعات عمومی و یادآمد خوراک براي هر فرد | |  | |  | ٭ | ٭ | ٭ | ٭ | ٭ | ٭ | ٭ | ٭ | ٭ | ٭ | ٭ | ٭ |  |  |  |  |
| 3 | | استخراج و آناليز آماری داده ها و تهيه گزارش نهايی | |  | |  |  |  |  |  |  |  |  |  |  |  |  |  | ٭ | ٭ | ٭ | ٭ |
| 4 | | پايش (Monitoring) | | ٭ | | ٭ | ٭ | ٭ | ٭ | ٭ | ٭ | ٭ | ٭ | ٭ | ٭ | ٭ | ٭ | ٭ | ٭ | ٭ | ٭ | ٭ |

**16- جدول بودجه بندي طرح**

*1 –* نيروي انساني

| *رديف* | *نوع فعاليت* | *عنوان شغل* | *تعداد افراد* | *ساعات مورد نياز* | *حق الزحمه در ساعت* | *جمع*  *(ريال)* |
| --- | --- | --- | --- | --- | --- | --- |
|  |  |  |  |  |  |  |
|  |  |  |  |  |  |  |
|  | | | | | *جمع* |  |

*2 – هزينه آزمايشات و خدمات تخصصي كه توسط ديگر مؤسسات صورت مي گيرد:*

| *رديف* | *موضوع آزمايش يا خدمات تخصصي* | *مركز سرويس دهنده* | *تعداد كل دفعات* | *هزينه براي هر دفعه* | *جمع*  *(ريال)* |
| --- | --- | --- | --- | --- | --- |
|  |  |  |  |  |  |
|  |  |  |  |  |  |
|  | | | | *جمع* |  |

*3 – فهرست وسايل و مواد مصرفي كه بايد خريداري شود :*

| رديف | *نام دستگاه و يا مواد* | *شركت سازنده و يا فروشنده* | *كشور* | *آيا در ايران موجود است* | *تعداد لازم* | *قيمت واحد* | *قيمت كل*  *(ريال)* |
| --- | --- | --- | --- | --- | --- | --- | --- |
|  | کیت هموسیستیین |  |  |  | 5 | 5000000 | 25000000 |
|  | کیت فولات |  |  |  | 5 | 5000000 | 25000000 |
|  | 5 متیل تتراهیدروفولات |  |  |  | 15000 | 1300 | 19500000 |
|  | اسید فولیک |  |  |  | 15000 | 30 | 450000 |
|  | | | | | | *جمع* | *69950000* |

*4- فهرست وسايل و مواد غيرمصرفي كه بايد خريداري شود :*

| رديف | *نام دستگاه و يا مواد* | *شركت سازنده و يا فروشنده* | *كشور* | *آيا در ايران موجود است* | *تعداد لازم* | *قيمت واحد* | *قيمت كل*  *(ريال)* |
| --- | --- | --- | --- | --- | --- | --- | --- |
|  |  |  |  |  |  |  |  |
|  |  |  |  |  |  |  |  |
|  | | | | | | *جمع* |  |

*5 – هزينه مسافرت (در صورت لزوم):*

| رديف | *مقصد* | *تعداد مسافرت در مدت اجراي طرح و منظور آن* | *نوع وسيله نقليه* | *تعداد افراد* | *هزينه*  *(ريال)* |
| --- | --- | --- | --- | --- | --- |
|  |  |  |  |  |  |
| *جمع* | | | | |  |

*6 – هزينه هاي تكثير و تايپ:*

| *رديف* | *نوع هزينه* | *هزينه*  *(ريال)* |
| --- | --- | --- |
|  | *هزينه هاي تكثير* |  |
|  | *هزينه هاي تايپ* |  |
|  | *هزينه سه جلد صحافي گزارش نهايي* |  |
| *جمع* | |  |

*7 – ساير هزينه ها:*

| *رديف* | *نوع هزينه* | *هزينه*  *(ريال)* |
| --- | --- | --- |
|  | *هزينه هاي پيش‌بيني نشده* |  |
|  | *ساير* |  |
| *جمع* | |  |

*8– جمع هزينه هاي طرح :*

| *رديف* | *نوع هزينه* | *جمع*  *(ريال)* |
| --- | --- | --- |
|  | *جمع نيروي انساني(جدول شماره 1)* |  |
|  | *جمع هزينه هاي آزمايشات و خدمات تخصصي(جدول شماره 2)* |  |
|  | *جمع هزينه هاي وسايل و مواد مصرفي (جدول شماره 3)* | *69950000* |
|  | *جمع هزينه هاي وسايل و مواد غيرمصرفي (جدول شماره 4)* |  |
|  | *جمع هزينه هاي مسافرت (جدول شماره 5)* |  |
|  | *جمع هزينه‌هاي تكثير و تايپ (جدول شماره 6)* |  |
|  | *ساير هزينه ها (جدول شماره 7)* |  |
| *جمع كل* | | *69950000* |

**17- فرم اظهارنامه پژوهشگر و ارزيابي اخلاق در پژوهش**

اين فرم بايد توسط مجری/مجريان تكميل و همراه با طرح پژوهشی به كميته اخلاق در پژوهش انستيتو تحقيقات تغذيه‌ای و صنايع غذايی كشور ارائه شود.

موضوع پژوهش: مقایسه تاثیر اسید فولیک و 5 متیل تترا هیدروفولات بر سطح سرمی فولات، هموسیستئین و میزان سقط در زنان مبتلا به سقط مکرر

نام مجری/مجريان: دکتر آزیتا حکمت دوست، دکتر سهیلا عارفی، آقای فرهاد وحید

محل اجرای پژوهش: مرکز درمان فوق تخصصی ابن سینا

كليه موارد ذيل بايد دارای مدارك و مستندات لازم باشد.

|  |  | بله |  | خير | نظر كميته اخلاق در پژوهش انستيتو |
| --- | --- | --- | --- | --- | --- |
| 1- باورها، رفتارها، سنتهای جامعه و موازين شرعي رعايت شده است؟ |  | ■ |  |  | ............................................................... |
| 2- مطالب متعارف و غير زننده بكار برده نشده است؟ |  | ■ |  |  | ............................................................... |
| 3- در بازنگري مدارك رعايت صداقت و امانت شده است؟ |  | ■ |  |  | ............................................................... |
| 4- از منابع معتبر و مورد تأييد استفاده گرديده است؟ |  | ■ |  |  | ............................................................... |
| 5- از جديدترين روشهای پژوهش استفاده شده است؟ |  | ■ |  |  | ............................................................... |
| 6- از روشهايی كه سبب آسيب‌جسمی يا روحی مي‌شود، استفاده نمي‌گردد؟ |  | ■ |  |  | ............................................................... |
| 7- رعايت آزادی فردی آزمودنی يا بيمار شده است؟ |  | ■ |  |  | ............................................................... |
| 8- فرم رضايت آگاهانه تنظيم شده و به امضای افراد خواهد رسيد؟  (يك نسخه از فرم مزبور را ضميمه كنيد) |  | ■ |  |  | ............................................................... |
| 9- ضرر و زيان ناشی از پژوهش كمتر از منافع آن است؟ |  | ■ |  |  | ............................................................... |
| 10- خسارات ناشی از صدمات جسمی يا زيان مالی ناشی از پژوهش كه ممكن است ناخواسته به افراد مورد بررسی وارد شود، جبران خواهد شد؟ |  | ■ |  |  | ............................................................... |
| 11- حقوق افراد صغير و يا كسانی كه قيم لازم دارند حفظ شده است؟ |  | ■ |  |  | ............................................................... |
| 12- قرار گرفتن آزمودنی در گروه شاهد و يا تجويز دارونما از نظر اخلاقی مورد تأييد است و موجب محروميت وی از خدمات ضروری نمي‌گردد؟ |  | ■ |  |  | ............................................................... |
| 13- قرار گرفتن آزمودنی در گروه شاهد و استفاده از دارونما به اطلاع وی خواهد رسيد؟ |  | ■ |  |  | ............................................................... |
| 14- اطلاعات مربوط به افراد مورد بررسی محرمانه باقی خواهد ماند؟ |  | ■ |  |  | ............................................................... |
| 15- كليه اهداف و روشهای اجرای پژوهش، نوع مداخله احتمالی و نتايج حاصل از پژوهش به كميته اخلاق در پژوهش اعلام شده است؟ |  | ■ |  |  | ............................................................... |
| 16- كليه اهداف و روشهای اجرای پژوهش، نوع مداخله احتمالی و نتايج حاصل از آن به اطلاع آزمودنی رسيده است؟ |  | ■ |  |  | ............................................................... |
| 17- به آزمودنی در مورد حق انصراف و خروج از پژوهش اطلاع رسانی شده است؟ |  | ■ |  |  | ............................................................... |

امضاء مجری/مجريان ...................................... تاريخ ...................................

| - نظر نهايی كميته اخلاق در پژوهش انستيتو در مورد رعايت اصول اخلاقی در پژوهش:  در جلسه ........................... مورخ........................ كميته اخلاق مورد تاييد قرار گرفت. |  |
| --- | --- |

امضاء دبير كميته اخلاق در پژوهش انستيتو ...................................

**18- فرم رضايت آگاهانه**

دانشگاه علوم پزشکی و خدمات بهداشتی درمانی شهید بهشتی

دانشکده علوم تغذیه و صنایع غذایی

پروژه پژوهشی: مقایسه تاثیر اسید فولیک و 5 متیل تترا هیدروفولات بر سطح سرمی فولات، هموسیستئین و میزان سقط در زنان مبتلا به سقط مکرر

اينجانب ............. بدین وسیله موافقت خود را برای شرکت در پروژه تحقيقاتي مقایسه تاثیر اسید فولیک و 5 متیل تترا هیدروفولات بر سطح سرمی فولات، هموسیستئین و میزان سقط در زنان مبتلا به سقط مکرر اعلام می دارم. زنان در گروه دریافت کننده مکمل اسید فولیک روزانه 5 میلی گرم (یک قرص) مکمل اسید فولیک و زنان گروه دریافت کننده مکمل 5 متیل تتراهیدروفولات روزانه 5 میلی گرم (یک قرص) مکمل 5 متیل تتراهیدروفولات در طول این مطالعه 7 ماهه دریافت خواهند کرد. در شروع و هفته هشتم مطالعه و نیز هفته ششم، دوازدهم و بیستم حاملگی از تمامی زنان در حالت ناشتا 10 سی سی خون وریدی گرفته می شود. در پایان هفته هشتم مطالعه کلیه زنان دوباره ویزیت شده و پس از دریافت مکمل ها، از آنها خواسته می شود تا اقدام به حاملگی نمایند، سپس در هفته های ششم، دوازدهم و بيستم حاملگی یا زمان سقط، تمامی زنان دوباره ویزیت شده و پس از دریافت مکمل ها جمع آوری نمونه های خونی، اندازه گیری های تن سنجی شامل قد و وزن برای کلیه افراد مورد مطالعه انجام خواهد گرفت. پرسشنامه های اطلاعات عمومی و یادآمد خوراک افراد مورد مطالعه نیز توسط کارشناسان تغذیه تکمیل خواهد شد. در این پژوهش کلیه آزمایشات خون رایگان خواهد بود.

محرمانه بودن اطلاعات:

تمام اطلاعاتي كه از اينجانب گرفته خواهد شد در تمام مراحل تحقيق از جمله انتشار نتايج محرمانه باقي خواهد ماند و محققين خود را موظف به رعايت امانت در حفظ اطلاعات مربوط به افراد می­دانند. البته برای اینجانب این حق محفوظ می باشد که در صورت عدم تمایل، به همکاری خود در این پروژه خاتمه دهم و در صورت هر گونه ضرر و زیان نیز این موافقت نامه مانع از اقدام قانونی اینجانب در مقابل مجریان این پژوهش و دانشگاه نخواهد شد. شرکت داوطلبانه در این پژوهش می تواند در ارائه درمان مؤثرتر و کم عارضه در زنان مبتلا به سقط مکرر موثر باشد.

نام فرد مسئول پاسخگويي به سوالات افراد شرکت کننده در بررسی : فرهاد وحید

تلفن: 09124443243

اينجانب .............................. ضمن مطالعه و اطلاع دقيق از موارد فوق و پذيرش آنها، رضايت خود را جهت شرکت در طرح حاضر اعلام می دارم.

امضاء فردشرکت کننده دربررسی: تاريخ:....................

امضاء همسر فردشرکت کننده دربررسی: تاريخ:....................

امضاء مجری طرح: تاريخ:....................

نام و امضای فرد تحويل گيرنده فرم: تاريخ:....................

آدرس فرد شرکت کننده در پژوهش: ..............

تلفن منزل:..................... تلفن محل کار:................

**منابع:**

1) Sutterlin M, Bussen S, Ruppert D , Steck T. Serum levels of folate and cobalamin in women with recurrent spontaneous abortion. Human Reproduction 1997;12:2292–2296.

2) Cornel MC, Erickson JD. Comparison of national policies on periconceptional use of folic acid to prevent spina bifida and anencephaly (SBA). Teratology 1997;55:134–7.

3) Eprova AG. Metafolin: about the product. 2000. Internet: http://www.metafolin.com. Accessed May 2002.

4) Groen V, Moser R. Synthesis of optically pure diastereoisomers of reduced folates. Pteridines 1999;10:95–100.

5) Rao KA, Pillai JR. Recurrent Pregnancy Loss. J Indian Med assoc 2006;104:458-461.

6) Abir R, Ornoy A, Hur HB et al. The effects of sera from women with spontaneous abortion on the in vitro development of early somite stage rat embryos. Am. J. Reprod. Immunol. 1994; 32:73–81.

7) Bulletti C, Flamigni C, Giacomucci E. Reproductive failure due to spontaneous abortion and recurrent miscarriage. Hum. Reprod. Update 1996;2:118–136.

8) Ray JG, Laskin CA. Folic acid and homocyst(e)ine metabolic defects and the risk of placental abruption, pre-eclampsia and spontaneous pregnancy loss: a systematic review. Placenta 1999;20:519–29.

9) Vollset SE, Refsum H, Irgens LM, et al. Plasma total homocysteine, pregnancy complications, and adverse pregnancy outcomes: the Hordaland Homocysteine Study. Am J Clin Nutr 2000;71:962–8.

10) Brouwer DA,Welten HT, Reijngoud DJ, van Doormaal JJ, Muskiet FA. Plasma folic acid cutoff value, derived from its relationship with homocyst(e)ine. Clin Chem 1998;44:1545–50.

11) Lucock MD, Daskalakis I, Schorah CJ, et al. Folate-homocysteine interrelations: potential new markers of folate status. Mol Genet Metab 1999;67:23–35.

12) Ma J, Stampfer MJ, Hennekens CH, et al. Methylenetetrahydrofolate reductase polymorphism, plasma folate, homocysteine, and risk of myocardial infarction in US physicians. Circulation 1996;94:2410–6.

13) Jacques PF, Bostom AG,Williams RR, et al. Relation between folate status, a common mutation in methylenetetrahydrofolate reductase and plasma homocysteine concentrations. Circulation 1996;93:7–9.

14) Candito M, Magnaldo S, Bayle J, Dor JF, Gillet Y, Bongain A, Van Obberghen E. [Clinical B12 deficiency in one case of recurrent spontaneous pregnancy loss.](../../../../%5C%5Cpubmed%5C12964808) [Clin Chem Lab Med.](javascript:AL_get(this, 'jour', 'Clin Chem Lab Med.');) 2003;41:1026-7.

15) Zetterberg H, Regland B, PalmeÂr M, Ricksten A, Palmqvist L, Rymo L, Arvanitis DA, Spandidos DA, Blennow K. Increased frequency of combined methylenetetrahydrofolate reductase C677T and A1298C mutated alleles in spontaneously aborted embryos. European Journal of Human Genetics 2002;10:113- 118.

16) Fohr IP, Prinz-Langenohl R, Brönstrup A, et al. 5,10-Methylenetetrahydrofolate reductase genotype determines the plasma homocysteine-lowering effect of supplementation with 5-methyltetrahydrofolate or folic acid in healthy young women. Am J Clin Nutr 2002;75:275–82.

17) Frosst P, Blom HJ,Milos R et al: A candidate genetic risk factor for vascular disease: a common mutation in methylenetetrahydrofolate reductase. Nat Genet 1995;10:111- 113.

18) Sotalo PA, Wells GA, Donnelly JG. [Neonatal and fetal methylenetetrahydrofolate reductase genetic polymorphisms: an examination of C677T and A1298C mutations.](../../../../%5C%5Cpubmed%5C10958762) [Am J Hum Genet.](javascript:AL_get(this, 'jour', 'Am J Hum Genet.');) 2000;67:986-90.

19) Nelen WL, Blom HJ, Thomas CM, Steegers EA, Boers GH, Eskes TK. Methylenetetrahydrofolate reductase polymorphism affects the change in homocysteine and folate concentrations resulting from low dose folic acid supplementation in women with unexplained recurrent miscarriages. J Nutr 1998;128:1336 – 41.

20) Tempfer CB, Kurz C, Bentz EK, Unfried G, Walch K, Czizek U, Huber JC. A combination treatment of prednisone, aspirin,folate, and progesterone in women with idiopathic recurrent miscarriage: a matched-pair study. Fertil Steril 2006;86:145– 8.

21) Czeizel AE, Dudas I, Metneki J. Pregnancy outcomes in a randomized controlled trial of periconceptional multivitamin supplementation. Arch. Gynecol. Obstet 1994;55: 131–139.

22) Wald N, Sneddon J, Densem J et al. Prevention of neural tube defects: results of the Medical Research Council Vitamin Study. Lancet 1991; 338:131–137.

23) Amin AF, Shaaban OM, Bediawy MA. [N-acetyl cysteine for treatment of recurrent unexplained pregnancy loss.](../../../../%5C%5Cpubmed%5C18983759) [Reprod Biomed Online](javascript:AL_get(this, 'jour', 'Reprod Biomed Online.');) 2008;17:722-6.

24) Goddijn M, van der Veen F, Ankum WM, Bonsel GJ, Leschot NJ, Boer K. [No consensus on the definition, diagnosis and treatment of habitual abortion in the Netherlands](../../../../%5C%5Cpubmed%5C10347665). [Ned Tijdschr Geneeskd](javascript:AL_get(this, 'jour', 'Ned Tijdschr Geneeskd.');) 1999; 143:897-902.

25) Nelen WL, Blom HJ, Steegers EA, den Heijer M, Thomas CM, Eskes TK. [Homocysteine and folate levels as risk factors for recurrent early pregnancy loss.](../../../../%5C%5Cpubmed%5C10725483) [Obstet Gynecol.](javascript:AL_get(this, 'jour', 'Obstet Gynecol.');) 2000 Apr;95(4):519-24.

26) Homocysteine Lowering Trialists’ Collaboration. Lowering blood homocysteine with folic acid based supplements: meta-analysis of randomised trials. BMJ 1998;316:894–8.

27) Brouwer IA, van Dusseldorp M, Thomas CM, et al. Low-dose folic acid supplementation decreases plasma homocysteine concentrations: a randomized trial. Am J Clin Nutr 1999;69:99–104.

28) Brönstrup A, Hages M, Prinz-Langenohl R, Pietrzik K. Effects of folic acid and combinations of folic acid and vitamin B-12 on plasma homocysteine concentrations in healthy, young women. Am J Clin Nutr 1998;68:1104–10.

29) Dierkes J, Kroesen M, Pietrzik K. Folic acid and vitamin B6 supplementation and plasma homocysteine concentrations in healthy young women. Int J Vitam Nutr Res 1998;68:98–103.

30) Ward M, McNulty H, McPartlin J, Strain JJ, Weir DG, Scott JM. Plasma homocysteine, a risk factor for cardiovascular disease, is lowered by physiological doses of folic acid. QJM 1997;90:519–24.

31) O’Keefe CA, Bailey LB, Thomas EA, et al. Controlled dietary folate affects folate status in nonpregnant women. J Nutr 1995;125:2717–25.

32) Nadir Y, Hoffman R, Brenner B. Association of homocysteine, vitamin B12, folic acid,and MTHFR C677T in patients with a thrombotic event or recurrent fetal loss. Ann Hematol 2007; 86:35–40.

33) Scott JM,Weir DG. The methyl folate trap. A physiological response in man to prevent methyl group deficiency in kwashiorkor (methionine deficiency) and an explanation for folic acid-induced exacerbation of subacute combined degeneration in pernicious anaemia. Lancet 1981;2:337–40.

34) Weir DG, Scott JM. Brain function in the elderly: role of vitamin B12 and folate. Br Med Bull 1999;55:669–82.

35) Venn BJ, Green TJ, Moser R, Mann JI. Comparison of the effect of low-dose supplementation with L-5-methyltetrahydrofolate or folic acid on plasma homocysteine:a randomized placebo-controlled study. Am J Clin Nutr 2003;77:658–62.

36) Que´re I, Mercierb E, Janbon C, Mare`s P, Gris JC. Vitamin supplementation and pregnancy outcome in women with recurrent early pregnancy loss and hyperhomocysteinemia. Fertility and Sterility 2001;75;823-825.

37) Franc M¸ vanta O, Zittounb J, Vayletc C, Pernetd P, Millieza J. Low Vitamin B12 level as a risk factor for very early recurrent abortion. European Journal of Obstetrics & Gynecology and Reproductive Biology 2002;104:156–159.

38) Kumar KS, Govindaiah V, Naushad SE, Devi RR, Jyothy A. [Plasma homocysteine levels correlated to interactions between folate status and methylene tetrahydrofolate reductase gene mutation in women with unexplained recurrent pregnancy loss.](../../../../%5C%5Cpubmed%5C12623486) [J Obstet Gynaecol.](javascript:AL_get(this, 'jour', 'J Obstet Gynaecol.');) 2003 ;23:55-58.

39) Lohman TG. Anthropometric standardization reference manual. Champaign, IL: Human Kinetics1988; 28-80.

40) Rosner B. Fundamentas of Biostatistics. 4^th^ ed. Belmont: Duxbury Press;1995:283.

41) Hulley SB, Cummings SR, Browner WS, Grady D, Hearst N, Newman TB. Designing Clininical Research: An Epidemiology Approach.2^nd^ ed; Philadelphia: Lippincott Williams & Wilkins;2001: 30.

**20- پيوستـها**

*دانشگاه علوم پزشکی و خدمات بهداشتی درمانی شهید بهشتی*

*دانشکده علوم تغذیه و صنایع غذایی*

*پيوست 2*

*پرسشنامه اطلاعات عمومی*

*پروژه پژوهشی: مقایسه تاثیر اسید فولیک و 5 متیل تترا هیدروفولات بر سطح سرمی فولات، هموسیستئین و میزان سقط در زنان مبتلا به سقط مکرر*

*نام و نام خانوادگی: کد: نام مرکز:*

*تلفن منزل، محل کار یا تلفن همراه:*

*آدرس (محل سکونت):*

*پست الکترونیکی:*

*1- سن:*

*2- ميزان تحصيلات: الف - بی سواد ب – ابتدایی ج – راهنمایی د - متوسطه*

*ه- دیپلم و- فوق دیپلم ز – لیسانس ح- فوق لیسانس و بالاتر*

*3- شغل (در صورت داشتن شغل آزاد با ذکر نوع):*

*4-استعمال دخانیات:* *بلی خير*

*مقدار مصرف: مدت مصرف:*

*5-استعمال دخانیات اطرافیان (مانند همسر، پدر و یا دیگران): بلی خير*

*6- مصرف مکمل ها: بلی خير نوع مکمل مصرفی :*

*7- مصرف قهوه: بلی خير*

*مقدار مصرف (فنجان در روز):*

*8- سوابق ابتلا به بیماری (با ذکر نوع، سن ابتلا و مدت زمان ابتلا به بیماری):*

*9- سوابق داروهاي مصرفي (با ذکر نوع و مقدار مصرف دارو):*

*10- داشتن رژيم غذايی به هر دليل:* *بلی خير*

*11- تعداد سقط مکرر قبل از آغاز مطالعه:*

*12- تعداد زایمان زنده قبل از آغاز مطالعه (بار):*

*13- تعداد فرزندان(نفر):*

*14- وضعیت سقط کننده:*

*سقط کننده اولیه  سقط کننده ثانویه *

*15- تاریخ آخرین سقط:*

*تاریخ: امضاء:*

دانشگاه علوم پزشکی و خدمات بهداشتی درمانی شهید بهشتی

دانشکده علوم تغذیه و صنایع غذایی

پروژه پژوهشی: مقایسه تاثیر اسید فولیک و 5 متیل تترا هیدروفولات بر سطح سرمی فولات، هموسیستئین و میزان سقط در زنان مبتلا به سقط مکرر

پیوست3 پرسشنامه یادآمد خوراک

نام و نام خانوادگی: کد:

شروع مطالعه  هفته هشتم  هفته ششم حاملگی هفته دوازدهم حاملگی هفته بیستم حاملگی 

| وعده غذایی | نوع غذا و اجزاء تشکیل دهنده | مقدار مصرف | تبدیل به گرم | ملاحظات |
| --- | --- | --- | --- | --- |
| صبحانه |  |  |  |  |
| میان وعده صبح |  |  |  |  |
| ناهار |  |  |  |  |
| میان وعده عصر |  |  |  |  |
| شام |  |  |  |  |
| قبل از خواب |  |  |  |  |

نام و نام خانوادگی پرسشگر: تاریخ: امضاء:
